# The circadian clock module LgPRR7–LgFKF1 negatively regulates flowering time in *Luculia gratissima*, a woody ornamental plant

**DOI:** 10.1093/hr/uhaf110

**Published:** 2025-04-24

**Authors:** Xiongfang Liu, Youming Wan, Jihua Wang, Fu Gao, Zihuan Wu, Zhenghong Li, Yao Zhang, Yongpeng Ma, Hong Ma

**Affiliations:** Institute of Highland Forest Science, Chinese Academy of Forestry, State Key Laboratory of Efficient Production of Forest Resources, Kunming 650216, China; Yunnan Key Laboratory for Integrative Conservation of Plant Species with Extremely Small Populations, Kunming Institute of Botany, Chinese Academy of Sciences, Kunming 650201, China; Institute of Highland Forest Science, Chinese Academy of Forestry, State Key Laboratory of Efficient Production of Forest Resources, Kunming 650216, China; Flower Research Institute, Yunnan Academy of Agricultural Sciences, Kunming 650205, China; National Engineering Research Center for Ornamental Horticulture, Kunming 650205, China; Yunnan Key Laboratory for Integrative Conservation of Plant Species with Extremely Small Populations, Kunming Institute of Botany, Chinese Academy of Sciences, Kunming 650201, China; General Work Station of Forest Seedlings of Yunnan Province, Kunming 650215, China; Institute of Highland Forest Science, Chinese Academy of Forestry, State Key Laboratory of Efficient Production of Forest Resources, Kunming 650216, China; Institute of Highland Forest Science, Chinese Academy of Forestry, State Key Laboratory of Efficient Production of Forest Resources, Kunming 650216, China; Yunnan Key Laboratory for Integrative Conservation of Plant Species with Extremely Small Populations, Kunming Institute of Botany, Chinese Academy of Sciences, Kunming 650201, China; Institute of Highland Forest Science, Chinese Academy of Forestry, State Key Laboratory of Efficient Production of Forest Resources, Kunming 650216, China; Key Laboratory of Breeding and Utilization of Resource Insects, National Forestry and Grassland Administration, Kunming 650233, China; Yunnan Key Laboratory of Breeding and Utilization of Resource Insects, Kunming 650224, China

## Abstract

Photoperiod-dependent flowering is a critical trait in breeding for flowering time in woody ornamental plants. Circadian clocks are vital for the regulation of photoperiodic flowering in plants, but their molecular regulation pathways in woody perennials remain poorly explored. Here, we identified two circadian clock components LgPSEUDO-RESPONSE REGULATOR 7 (LgPRR7) and LgFLAVIN-BINDING KELCH REPEAT F-BOX 1 (LgFKF1) as key repressors of flowering in *Luculia gratissima*, a short-day woody ornamental plant with commercial potential. Levels of *LgPRR7* and *LgFKF1* transcripts exhibited photoperiodic responses and diurnal patterns. Ectopic overexpression of *LgPRR7* or *LgFKF1* in *Arabidopsis thaliana* accelerated flowering, whereas silencing *LgPRR7* or *LgFKF1* in *L. gratissima* accelerated flowering. Crucially, LgPRR7 directly interacts with LgFKF1, forming a self-reinforcing regulatory module LgPRR7–LgFKF1 to repress flowering in *L. gratissima*. Furthermore, the observed physical interactions among LgFKF1, LgCONSTANS-LIKE 12 (LgCOL12), and LgREPRESSOR OF ga1–3-LIKE 2 (LgRGL2) implied that they possibly formed a protein complex LgFKF1–LgCOL12–LgRGL2, bridging the circadian clock, photoperiod, and gibberellin signaling pathways to suppress downstream floral integrators. Intriguingly, silencing *LgPRR7* and *LgFKF1* extended the duration of *L*. *gratissima* flowering, a trait of horticultural significance. These results suggest the integration of multilayered environmental and endogenous signals in the regulation of flowering time. The LgPRR7–LgFKF1 module provides novel targets for molecular improvement to manipulate flowering time and duration in *L. gratissima* and other economically valuable woody ornamental plants. Our results also support the mediation of flowering convergence in short-day plants through the action of circadian clock genes.

## Introduction

In angiosperms, the precise regulation of flowering time is critical for ensuring reproductive success [1]. This process is controlled by internal and external factors, of which the photoperiod is a key environmental factor [1, 2]. Studies into the molecular regulatory networks underlying photoperiod-induced flowering have been conducted in the model plant Arabidopsis, among others [3–7]. In the photoperiod flowering pathway, plants receive light and circadian signals and integrate these inputs to modulate the expression of *CONSTANS* (*CO*) and *FLOWERING LOCUS T* (*FT*) [6]. Circadian clock genes play a pivotal role in the photoperiod pathway, and nearly all Arabidopsis circadian clock genes are involved in flowering regulation [6]. However, the molecular networks controlling flowering time through the circadian clock are functionally divergent across species [6]. In Arabidopsis, the components of evening complex (EC), which include *EARLY FLOWERING 3* (*ELF3*), *ELF4*, and *LUX ARRHYTHMO* (*LUX*), act as floral repressors [8–11]. The EC orthologs also function as floral repressors in barley (*Hordeum vulgare*) [12, 13], pea (*Pisum sativum*) [14–16], and *Medicago truncatula* [17]; however, their orthologs *OsELF3/4a* and *OsLUX* in rice (*Oryza sativa*) [18, 19] and *GmELF3* and *GmLUX* in soybean (*Glycine max*) [20, 21] act as floral activators. Members of the PSEUDO-RESPONSE REGULATOR (PRR) family, including PRR3/5/7/9 and TOC1 in Arabidopsis, are key components of the central oscillator of the plant circadian clock [22]. PRR5, PRR7, and PRR9 have been found to positively control flowering in Arabidopsis [23, 24]. The *PRR7* orthologs *HvPpd-H1* in barley and *BvBTC1* in sugar beet (*Beta vulgaris*) act as floral activators [25, 26], whereas *GmPRR37* in soybean and *OsPRR73* in rice function as inhibitors [27, 28]. FLAVIN-BINDING KELCH REPEAT F-BOX 1 (FKF1), a circadian-regulated blue light receptor and component of the SKP1/CUL1/F-box (SCF) E3 ligase complex [29–31], accelerates flowering in Arabidopsis [32] but delays it in soybean via *GmFKF1a/b* [33]. Although the circadian clock genes are essential for photoperiodic flowering in plants, their regulatory networks in woody perennial plants remain poorly understood. As critical components of the circadian clock, *PRR7* and *FKF1* play roles in modulating plant photoperiod flowering [23–34]. However, whether FKF1 interacts with PRR7 to regulate flowering has not yet been reported, even in model plants.

*Luculia gratissima* is a small tree or shrub in the Rubiaceae, distributed along the southeastern edge of the Tibetan Plateau in Southwest China and the neighboring regions of Nepal and Myanmar [35]. It has good ornamental characteristics, including a large, dense inflorescence with pink flowers, a rich, sweet floral fragrance, a long flowering period, and evergreen leaves. It can be cultivated as a garden plant, a potted plant, or to produce cut flowers. Because of this, *L. gratissima* is an important horticultural flower plant with great ornamental value and potential for further economic development. *L. gratissima* ‘Xiangfei’, a cultivar with strong disease resistance and cold resistance, is a typical obligate short-day plant with a flowering period concentrated in the autumn and winter seasons in natural conditions [36]. However, the molecular mechanisms underlying regulation of *L. gratissima* ‘Xiangfei’ flowering remain unexplored. The high energy costs associated with year-round production through traditional flowering-time control techniques have severely constrained the sustainable commercial production of *L. gratissima*. Thus, identifying key flowering regulators in this plant will contribute to its molecular improvement and breeding efforts focused on flowering time, and promote its year-round production and supply.

In this study, we characterized the morphological, physiological, and transcriptional changes in *L. gratissima* following treatment with different photoperiods. The clock genes *LgPRR7* and *LgFKF1* were identified in *L. gratissima* and their expression patterns were examined during flowering. Subsequently, *LgPRR7* and *LgFKF1* were functionally characterized in *Arabidopsis thaliana* and *L. gratissima*. Additionally, we found that the endogenous gibberellin (GA)_7_ responded to photoperiods during flowering in *L. gratissima*, and we confirmed that the interaction between LgPRR7 and LgFKF1 suppressed flowering in *L. gratissima* synergistically. The LgPRR7–LgFKF1 module affected the flowering-related genes *LgFT*, *LgLEAFY* (*LgLFY*), and *LgAPETALA1* (*LgAP1*) via the LgFKF1–LgCOL12–LgRGL2 complex, with GA_7_ potentially participating in this process through contact with LgRGL2. These findings provide molecular evidence that the LgPRR7–LgFKF1 module mediates flowering in *L. gratissima*.

## Results

### Morphological development, changes in endogenous GA levels, and networks regulating transcription during flowering in *L. gratissima*

Morphoanatomical results showed that the developmental stages of apical buds in *L. gratissima* at 10, 20, and 30 days following the controlled short-day (SH, 10-hour light /14-hour dark at 20°C) treatment corresponded to the differentiation of inflorescence primordia, perianth primordia, and pistil primordia, respectively (Fig. S1A–F). The apical buds remained in the vegetative phase throughout all developmental stages when the plants were grown under controlled long-day (LH, 12-hour light/12-hour dark with a 4-hour night break at 20°C) conditions (Fig. S1G–L).

In the apical buds and leaves of *L. gratissima*, levels of bioactive GA_7_ showed greater differences between the SH and LH treatments than did other bioactive GAs, including GA_1_, GA_3_, and GA_4_ (Fig. S2A). Next, we aimed to identify key GAs and related genes in the GA regulatory pathways during flowering in *L. gratissima* grown under different photoperiod treatments. We screened GAs and their corresponding gene modules using the module-GA correlation analysis, with a threshold value of |cor| ≥ 0.6 and *P* < 0.05, and we identified a total of six GAs and eight corresponding gene modules (Fig. S2B). Notably, the bioactive GA_3_ (cor = 0.99) and GA_7_ (cor = 0.73) showed strongly positive correlations with *GIBBERELLIN 20-OXIDASE* (*GA20OX1*) (Fig. S2C; Table S1), a gene of the GA biosynthesis pathway [3], and the dynamic changes in GA_3_ and GA_7_ content (Fig. S2A) exhibited temporal consistency with the expression patterns of *GA20OX1* (Fig. S2D). The GA signal transduction pathway is initiated through the binding of bioactive GAs to the receptor GA-INSENSITIVE DWARF 1 (GID1) [3]. In our study, GA_7_ showed stronger correlation with the upregulated GA receptor gene *GID1B* (cor = 0.69) than did GA_3_ (cor = 0.58) (Fig. S2C; Table S1), which was consistent with the results of the analysis of GA levels (Fig. S2A). Moreover, the downregulated GA signal suppressor gene *RGL2* was negatively correlated with GA_3_ and GA_7_, and the degree of negative correlation was higher in GA_7_ (cor = −0.55) than in GA_3_ (cor = −0.52) (Fig. S2C; Table S1). These findings collectively identify GA_7_ as one of the key bioactive GAs regulating flowering in *L. gratissima*.

A total of 2765 differentially expressed genes (DEGs) were identified in the three comparisons (SH10 vs. LH10, SH20 vs. LH20, and SH30 vs. LH30) (Fig. S3A–D; Table S2). These DEGs were mainly enriched in the pathways plant circadian rhythm, DNA replication, and plant hormone signal transduction (Fig. S3E). Furthermore, we identified 30 DEGs involved in key flowering pathways, including the photoperiod pathway, the GA pathway, floral integrator genes, and meristem identity genes (Fig. S3F). The reliability and validity of the RNA-sequencing (RNA-seq) data were validated using quantitative reverse transcription–polymerase chain reactions (RT-qPCR) (Fig. S4).

The weighted gene co-expression network analysis (WGCNA) identified genes with correlated expression patterns (Fig. S5A), which may function in shared pathways. However, transcriptional co-expression of the genes does not necessarily imply physical interaction or direct regulatory relationships. Protein–protein interaction (PPI) networks complement transcriptomic data by revealing potential molecular partnerships that underlie biological processes. The PPI network derived from the genes in the WGCNA modules (Fig. S5A; Table S3) revealed potential regulatory relationships among the circadian clock, GA signaling, and photoperiodic flowering pathways in *L. gratissima* (Fig. S5B). Notably, the clock proteins LgPRR7 and LgFKF1 were putatively co-expressed within the PPI network (Fig. S5C), indicating a potential interaction relationship between the clock LgPRR7 and LgFKF1, which warrants further investigation.

### Identification of the clock genes *LgPRR7* and *LgFKF1* in *L. gratissima*

Previous RNA-seq data showed that short-day conditions suppressed *LgPRR7* and *LgFKF1* expression in *L. gratissima* [37]. Similarly, in this study, the clock genes *LgPRR7* and *LgFKF1* were significantly downregulated in *L. gratissima* at 10 and 20 days, respectively, postinitiation of SH treatment compared with the LH treatment (Table S2). In addition, a putative co-expression relationship between LgPRR7 and LgFKF1 was observed in the PPI network (Fig. S5C). These findings suggest that the clock genes *LgPRR7* and *LgFKF1* might be essential for flowering in *L. gratissima*.

The full-length cDNA sequences of *LgPRR7* and *LgFKF1* in *L. gratissima* and their orthologous sequences in *A. thaliana* shared 70.54 and 74.12% sequence similarity, respectively (Table S4). The protein LgPRR7 contains the psREC_PRR and CCT conserved domains (Fig. S6), whereas LgFKF1 contains the LOV (PAS), F-box, and Kelch repeat domains (Fig. S7). Phylogenetic analyses showed that compared to the orthologous proteins AtPRR7 or AtFKF1 in *A. thaliana*, LgPRR7 shared greater similarity with *Coffea* orthologous protein sequences (Fig. S8A), whereas LgFKF1 exhibited higher similarity to orthologous protein sequences in other Rubiaceae species (Fig. S8B). It is of note that multiple light-responsive *cis*-acting elements were predicted in the promoter sequences of *LgPRR7* and *LgFKF1* (Fig. S9A), along with abundant potential transcription binding sites implicated in photoperiod regulation, circadian clock, and GA signal transduction (Fig. S9B).

**Figure 1:**
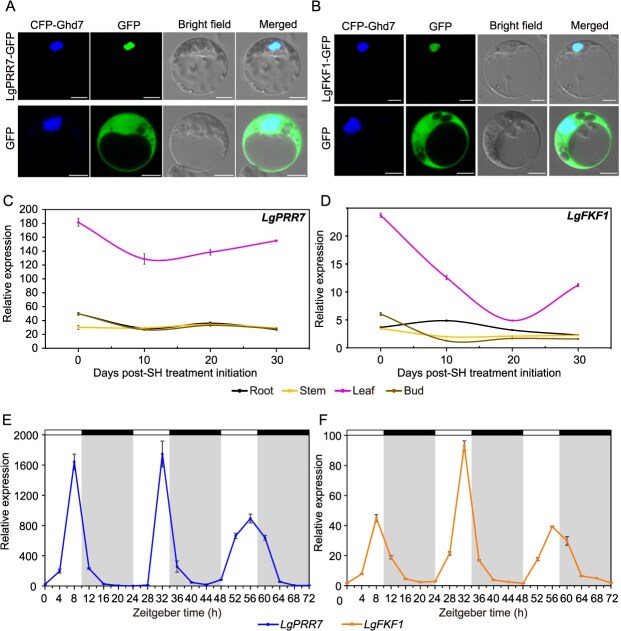
Subcellular localization of proteins LgPRR7 and LgFKF1, and spatiotemporal and diurnal expression of *LgPRR7* and *LgFKF1* in *Luculia gratissima*. **(A** and **B)** Transient expression of *35S*::*LgPRR7*-*GFP***(A)** and *35S*::*LgFKF1*-*GFP***(B)** in *Arabidopsis thaliana* protoplasts. Scale bar: 5 μm. **(C** and **D)** Spatiotemporal expression patterns of *LgPRR7***(C)** and *LgFKF1***(D)** in the roots, stems, leaves, and buds of *L. gratissima* at four times following initiation of SH treatment. Mean values ± standard error are shown from three biological replicates (*n* = 3). **(E** and **F)** Diurnal expression patterns of *LgPRR7***(E)** and *LgFKF1***(F)** in *L. gratissima* leaves over a 72-hour period (three consecutive 10-hour light/14-hour dark cycles) starting from 30 days post-SH treatment. Mean values ± standard error are shown from three biological replicates (*n* = 3). White and gray (or black) areas (or bars) represent light and dark phases, respectively. SH: the controlled short-day treatment (10-hour light from 8:00 to 18:00/14-hour dark at 20°C). Zeitgeber time (ZT) is defined as ZT0 being lights-on.

Subcellular localization analysis indicated overlapping signals of 35S::LgPRR7-GFP or 35S::LgFKF1-GFP with the nuclear marker 35S::Ghd7-CFP (Fig. 1A and B), confirming the nuclear localization of LgPRR7 and LgFKF1. In *L. gratissima*, *LgPRR7* and *LgFKF1* were mainly expressed in the leaves, and showed an initial decrease followed by an increase in expression during development, reaching their lowest levels 10 and 20 days post-SH treatment initiation, respectively (Fig. 1C and D). The 72-hour time-course analysis spanning three consecutive light–dark (10-hour light/14-hour dark) cycles revealed diurnal expression patterns of *LgPRR7* and *LgFKF1* transcripts in SH-treated leaves, with peaks consistently occurring before the end of the light phase in each light–dark cycle (Fig. 1E and F).

### Ectopic overexpression of *LgPRR7* in *A. thaliana* accelerated flowering

**Figure 2:**
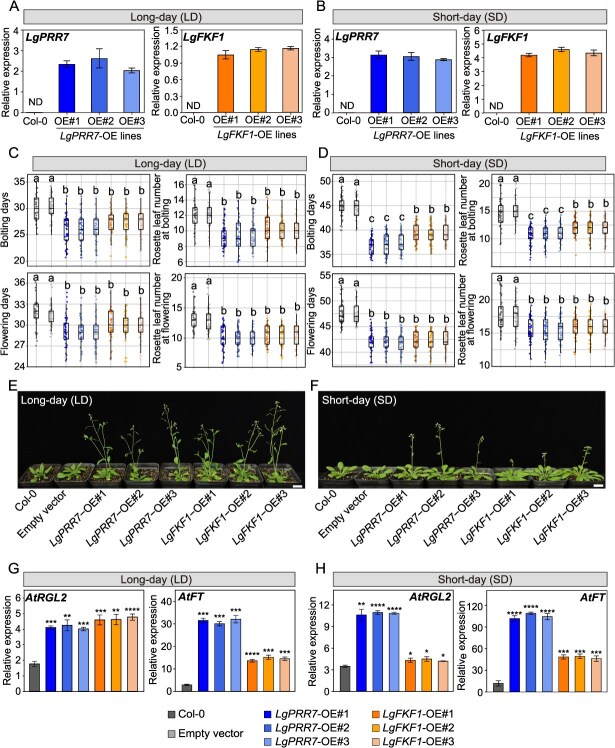
Ectopic overexpression of *LgPRR7* or *LgFKF1* in *Arabidopsis thaliana*. **(A** and **B)** RT-qPCR analyses of *LgPRR7* and *LgFKF1* transcript levels in *LgPRR7*-OE and *LgFKF1-*OE transgenic lines at the four-leaf stage under LD **(A)** and SD **(B)** conditions. *LgPRR7*- and *LgFKF1*-OE#1, #2, and #3 correspond to three independent *LgPRR7*- and *LgFKF1*-overexpressing transgenic lines, respectively. LD: controlled long-day (16-hour light from 6:00 to 22:00 /8-hour dark at 22°C); SD: controlled short-day (8-hour light from 8:00 to 16:00/16-hour dark at 22°C). Mean ± standard deviation are shown from three biological replicates (*n* = 3). ND: not detected. **(C** and **D)** Statistical data for days to bolting and flowering as well as rosette leaf number at the bolting and flowering stages of *LgPRR7*- and *LgFKF1*-overexpressing transgenic *A. thaliana* lines under LD **(C)** and SD **(D)** conditions. Different lowercase letters indicate statistically significant differences (*n* ≥ 25; Scheffe’s test: *P* < 0.05). **(E** and **F)** Flowering phenotype of *LgPRR7*- and *LgFKF1*-overexpressing transgenic *A. thaliana* lines under LD **(E)** and SD **(F)** treatments. Scale bar: 1 cm. **(G** and **H)** RT-qPCR analyses of *AtRGL2* and *AtFT* transcript levels in *LgPRR7*-OE and *LgFKF1-*OE transgenic lines during the flowering initiation stage under LD **(G)** and SD **(H)** conditions. Mean ± standard deviation are shown from three biological replicates (*n* = 3). Asterisks indicate values that are statistically different from those of the wild-type Col-0 control (Student’s *t* test; *P < 0.05, **P < 0.01, ***P < 0.001, ****P < 0.0001, ns: not significant).

To assess whether *LgPRR7* regulates flowering time in plants, *LgPRR7* was overexpressed (OE) in *A. thaliana*. A total of six independent *T*_4_ transgenic *A. thaliana* lines (*LgPRR7*-OE) were obtained (Fig. S10A). Three *LgPRR7*-OE lines (OE#1, OE#2, and OE#3) were selected for further functional analysis. RT-qPCR analysis confirmed that *LgPRR7* expression levels were considerably higher in the three *LgPRR7*-OE lines than that in the wild-type Col-0 plants (Fig. 2A and B). There were no significant differences between wild-type Col-0 and empty vector (pCAMBIA1300S) controls of *A. thaliana* in bolting and flowering phenotypes (Fig. 2C–F), indicating that the pCAMBIA1300S vector had no effect on the growth and development of *A. thaliana* and that wild-type Col-0 plants could serve as a control for transgenic lines.

Under the controlled long-day (LD, 16-hour light/8-hour dark at 22°C), the bolting time of *LgPRR7*-overexpressing transgenic lines *LgPRR7*-OE#1 (25.96 ± 2.95 days), -OE#2 (25.84 ± 2.94 days), and -OE#3 (26.03 ± 2.60 days) was found to be approximately 4 days earlier than that of the wild-type Col-0 controls (30.06 ± 2.51 days), and *LgPRR7*-OE lines had significantly fewer rosette leaves at bolting (Fig. 2C). Furthermore, the *LgPRR7*-OE lines *LgPRR7*-OE#1 (29.25 ± 2.17 days), -OE#2 (29.36 ± 1.97 days), and -OE#3 (29.07 ± 2.22 days) flowered 2–3 days earlier compared to the Col-0 controls (31.59 ± 2.08 days), with significantly fewer rosette leaves at flowering (Fig. 2C and E).

Similarly, under the controlled short-day (SD, 8-hour light/16-hour dark at 22°C) condition, the *LgPRR7*-OE lines *LgPRR7*-OE#1 (36.96 ± 1.74 days), -OE#2 (37.11 ± 2.20 days), and -OE#3 (37.16 ± 1.97 days) bolted approximately 8 days earlier compared to the Col-0 controls (44.82 ± 2.17 days), with considerably fewer rosette leaves at bolting (Fig. 2D). Moreover, compared with the Col-0 controls (47.58 ± 2.12 days), *LgPRR7*-OE lines OE#1 (42.12 ± 2.01 days), -OE#2 (41.98 ± 1.89 days), and -OE#3 (42.02 ± 1.96 days) flowered 5–6 days earlier than the controls, with significantly fewer rosette leaves at flowering (Fig. 2D and F).

We then analyzed the expression levels of flowering-related genes in *LgPRR7*-OE transgenic lines. Under both LD and SD conditions, the levels of *AtFT* and *AtRGL2* transcripts were significantly upregulated in the *LgPRR7*-OE transgenic lines compared to the Col-0 controls (Fig. 2G–H). These results indicate that *LgPRR7* overexpression in *A. thaliana* significantly promoted flowering and affected the expression of flowering-related genes.

### Silencing of *LgPRR7* accelerated flowering in *L. gratissima*

**Figure 3:**
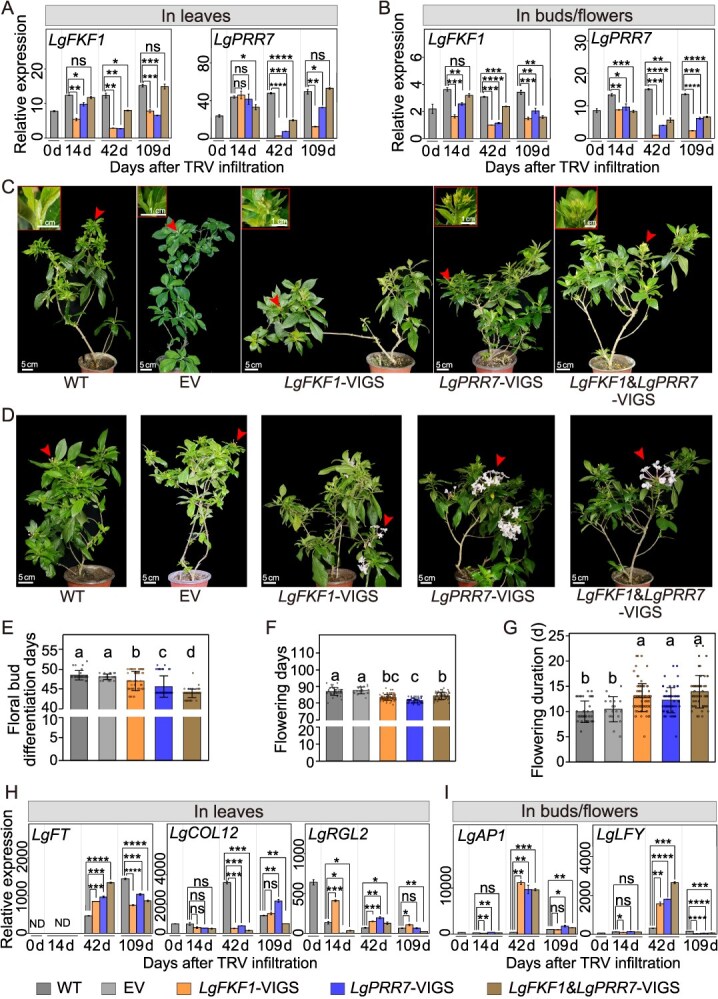
Effects of silencing *LgPRR7* and *LgFKF1* on *Luculia gratissima.***(A** and **B)** RT-qPCR analyses of levels of *LgPRR7* and *LgFKF1* transcripts in leaves **(A)** and buds/flowers **(B)** of *LgPRR7*-VIGS, *LgFKF1*-VIGS, and *LgFKF1&LgPRR7*-VIGS *L. gratissima* plants under SH conditions. *LgPRR7*-VIGS, *LgFKF1*-VIGS, and *LgFKF1&LgPRR7*-VIGS represent *L. gratissima* plants infiltrated with the VIGS constructs pTRV-*LgPRR7*, pTRV-*LgFKF1*, and pTRV-*LgFKF1&LgPRR7*, respectively; WT and EV represent wild-type plants (WT; non-infiltrated) and pTRV empty vector controls, respectively. SH: controlled short-day (10-hour light from 8:00 to 18:00/14-hour dark at 20°C). Mean ± standard deviation are shown from three biological replicates (*n* = 3). **(C** and **D)** Phenotypes of floral bud differentiation **(C)** and flowering **(D)** in *LgPRR7*-VIGS, *LgFKF1*-VIGS, and *LgFKF1&LgPRR7*-VIGS *L. gratissima* plants under SH conditions. Scale bar: 5 cm. **(E** and **G)** Statistical data of days to floral bud differentiation **(E)**, days to flowering **(F)**, and flowering duration **(G)**. Mean ± standard deviation are shown (*n* ≥ 25). Different lowercase letters indicate statistically significant differences (Scheffe’s test: *P* < 0.05). **(H** and **I)** RT-qPCR analyses of levels of *LgFT*, *LgCOL12*, and *LgRGL2* transcripts in leaves **(H)** and *LgAP1* and *LgLFY* transcripts in buds/flowers **(I)** of *LgPRR7*-VIGS, *LgFKF1*-VIGS, and *LgFKF1&LgPRR7*-VIGS *L. gratissima* plants under SH conditions. Mean ± standard deviation are shown from three biological replicates (*n* = 3). Asterisks indicate values that are statistically different from the WT controls (Student’s *t* test; *P < 0.05, **P < 0.01, ***P < 0.001, ****P < 0.0001, ns: not significant). ND: not detected.

To clarify the effect of *LgPRR7* on floral development in *L. gratissima*, *LgPRR7*-silenced (*LgPRR7*-virus-induced gene silencing [VIGS]) *L. gratissima* plants were generated using a tobacco rattle virus (TRV)-based-VIGS system. Given that *L. gratissima* is an obligate short-day plant [36], and infiltrated *L. gratissima* plants maintained vegetative growth under LH conditions, subsequent analyses were only conducted under SH conditions. Compared to the non-infiltrated plants (wild-type controls), the levels of *LgPRR7* transcripts in *LgPRR7*-silenced (*LgPRR7*-VIGS) *L. gratissima* plants were significantly downregulated 42 days after TRV infiltration (Fig. 3A and B). There were no significant phenotypic differences between the wild-type (WT) and pTRV empty vector (EV) controls of *L. gratissima* plants (Fig. 3C–G), indicating that the pTRV vector had no effect on the growth and development of *L. gratissima* plants and that the WT (non-infiltrated) plants could serve as a control for the VIGS *L. gratissima* plants. The floral bud differentiation in *LgPRR7*-VIGS plants (45.56 ± 2.70 days) was observed to occur approximately 3 days earlier than that in the WT controls (48.45 ± 1.21 days) (Fig. 3C and E). Furthermore, compared with the WT controls (87.00 ± 2.38 days), *LgPRR7*-VIGS plants (81.56 ± 1.42 days) flowered approximately 5 days earlier (Fig. 3D and F). Interestingly, the flowering duration of *LgPRR7*-VIGS plants was extended significantly compared to that of the WT controls (12.21 ± 2.50 days vs. 9.93 ± 2.14 days), representing an approximately two-day prolongation (Fig. 3G). Finally, we analyzed the expression levels of flowering-related genes in *LgPRR7*-VIGS *L. gratissima* plants. Compared with the WT controls, *LgPRR7*-VIGS *L. gratissima* plants exhibited substantially higher expression of *LgFT* and *LgRGL2* in the leaves (Fig. 3H) and higher expression of *LgAP1* and *LgLFY* in the buds or flowers (Fig. 3I), but significantly lower expression of *LgCOL1*2 in the leaves (Fig. 3H), at 42 days after TRV infiltration. These results indicate that *LgPRR7* is involved in regulating floral bud differentiation and flowering in *L. gratissima*, and influences the flowering duration.

### The circadian clock component LgPRR7 directly interacted with LgFKF1

**Figure 4:**
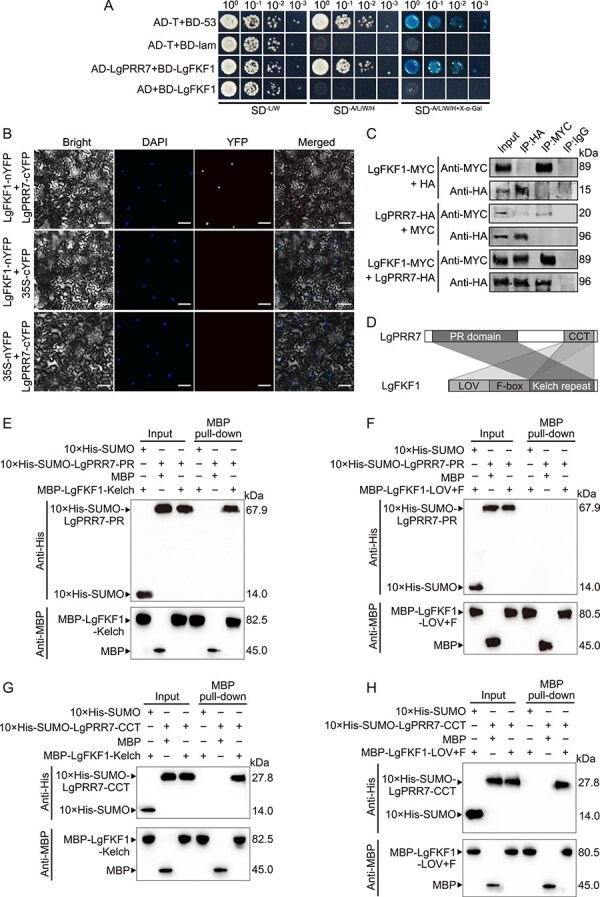
LgPRR7 interacts with LgFKF1. **(A)** Yeast two-hybrid assay showing the interaction of LgPRR7 and LgFKF1 in yeast. Yeast was spotted onto a synthetic double dropout (−L/W), a quadruple dropout (−A/L/W/H), or a quadruple dropout supplemented with X-α-Gal (−A/L/W/H + X-α-Gal) medium. A (Adenine), L (Leucine), W (Tryptophan), H (Histidine). BD, bait protein fused to GAL4 DNA-binding domain; AD, prey protein fused to GAL4 activation domain. The combinations pGADT7-T + pGBKT7–53 and pGADT7-T + pGBKT7-lam were used as the positive and negative controls, respectively. **(B)** Bimolecular fluorescence complementation (BiFC) assay showing the interaction of LgPRR7 and LgFKF1 in tobacco leaves. The nuclei are indicated by 4,6-diamidino-2-phenylindole dihydrochloride (DAPI) staining. Scale bar: 25 μm. **(C)** Co-immunoprecipitation (Co-IP) assay showing the interaction of LgPRR7 and LgFKF1 in tobacco leaves. Protein samples were extracted from tobacco leaves transiently expressing LgFKF1-MYC and LgPRR7-HA. Immunoprecipitates obtained with anti-MYC or anti-HA magnetic microbeads were analyzed using immunoblotting with anti-MYC and anti-HA antibodies, respectively. Input (crude extracts) were used as a positive control to determine whether the target protein was expressed. IgG antibody was used as a negative control to exclude potential non-specific binding between the target proteins and antibodies. **(D)** Diagrams depicting the linear structures of LgPRR7 and LgFKF1. PR domain: LgPRR7 N-terminal PR domain; CCT: LgPRR7 C-terminal CCT domain. LOV: LgFKF1 N-terminal LOV domain; F-box: LgFKF1 F-box domain; Kelch repeat: LgFKF1 C-terminal Kelch repeat domain. **(E)***In vitro* pull-down assay showing the direct interaction of the LgPRR7 PR domain with the LgFKF1 Kelch repeat domain. Recombinant 10 × His-SUMO-LgPRR7-PR was incubated with immunoprecipitated MBP-LgFKF1-Kelch. **(F)***In vitro* pull-down assay showing no detectable interaction between the LgPRR7 PR domain and the LOV or F-box domains of LgFKF1. Recombinant 10 × His-SUMO-LgPRR7-PR was incubated with immunoprecipitated MBP-LgFKF1-LOV + F. **(G)***In vitro* pull-down assay showing the direct interaction of the LgPRR7 CCT domain with the LgFKF1 Kelch repeat domain. Recombinant 10 × His-SUMO-LgPRR7-CCT was incubated with immunoprecipitated MBP-LgFKF1-Kelch. **(H)***In vitro* pull-down assay showing the direct interaction of the LgPRR7 CCT domain with the LOV and F-box domains of LgFKF1. Recombinant 10 × His-SUMO-LgPRR7-CCT was incubated with immunoprecipitated MBP-LgFKF1-LOV + F. LgPRR7-PR in **(E** and **F)** represents the LgPRR7 PR domain; LgPRR7-CCT in **(G** and **H)** represents the LgPRR7 CCT domain; LgFKF1-Kelch in **(E** and **G)** represents the LgFKF1 Kelch repeat domain; LgFKF1-LOV + F in **(F** and **H)** represents the LOV and F-box domains of LgFKF1. The “+” or “−” in (**E–H**) represent the presence or absence of proteins, respectively. Proteins in **(E–H)** were detected using immunoblotting with anti-His or anti-MBP antibodies.

To further investigate the regulatory role of the clock protein LgPRR7 in flowering, we selected the clock-related protein LgFKF1, which is potentially co-expressed with LgPRR7 in the flowering regulatory network (Fig. S5C). To examine this possibility, we first assessed the interaction between LgPRR7 and LgFKF1 with a yeast two-hybrid assay and found that LgPRR7 interacted with LgFKF1 (Fig. 4A). The interaction *in planta* between LgPRR7 and LgFKF1 was further confirmed using bimolecular fluorescence complementation (BiFC) and co-immunoprecipitation (Co-IP) assays. More specifically, the BiFC results revealed strong reconstituted yellow fluorescent protein signals in the nuclei of tobacco leaf cells co-expressing LgPRR7-cYFP and LgFKF1-nYFP (Fig. 4B). In the Co-IP assay, LgPRR7-HA was precipitated with LgFKF1-MYC (Fig. 4C). We further confirmed the direct interaction between LgPRR7 and LgFKF1 using an *in vitro* pull-down assay (Fig. 4E–H). Our MBP pull-down analyses demonstrated that the N-terminal pseudoreceiver (PR) domain of LgPRR7 specifically interacted with the C-terminal Kelch repeats but not the N-terminal LOV or F-box domains of LgFKF1 (Fig. 4D–F). Furthermore, the C-terminal CCT domain of LgPRR7 showed an interaction not only with the Kelch repeats, but also with the LOV and F-box domains of LgFKF1 (Fig. 4D, G, and H). Together, these results indicated that LgPRR7 physically interacted with LgFKF1 *in vivo* and *in vitro*.

### Altered *LgFKF1* expression affected flowering time and flowering-related gene expression

The observed interaction between LgPRR7 and LgFKF1 prompted us to evaluate the role of *LgFKF1* in flowering. To investigate the relationship between *LgFKF1* expression and flowering time, we overexpressed *LgFKF1* in *A. thaliana*. A total of five independent *T*_4_ transgenic *A. thaliana* lines (*LgFKF1*-OE) were obtained (Fig. S10B). Three *LgFKF1*-OE lines (OE#1, OE#2, and OE#3) were selected for functional characterization. RT-qPCR analysis confirmed that the levels of *LgFKF1* transcripts in the three transgenic lines were all significantly increased compared with those in Col-0 plants (Fig. 2A and B). Under LD conditions, the *LgFKF1*-overexpressing transgenic lines (*LgFKF1*-OE#1, 27.36 ± 2.15 days; -OE#2, 27.24 ± 2.48 days; and -OE#3, 27.16 ± 2.25 days) bolted approximately 3 days earlier than did the wild-type Col-0 controls (30.06 ± 2.51 days), and *LgFKF1*-OE lines had significantly fewer rosette leaves at bolting compared to the Col-0 controls (Fig. 2C). Moreover, *LgFKF1*-OE lines *LgFKF1*-OE#1 (29.95 ± 2.22 days), -OE#2 (29.77 ± 2.36 days), and -OE#3 (29.89 ± 2.15 days) flowered approximately 2 days earlier compared to the Col-0 controls (31.59 ± 2.08 days), with significantly fewer rosette leaves at flowering (Fig. 2C and E).

Under SD conditions, *LgFKF1*-OE lines *LgFKF1*-OE#1 (39.42 ± 1.89 days), -OE#2 (38.89 ± 1.94 days), and -OE#3 (39.33 ± 1.91 days) bolted 5–6 days earlier compared to the Col-0 controls (44.82 ± 2.17 days), with considerably fewer rosette leaves at bolting compared to the Col-0 controls (Fig. 2D). Furthermore, compared with the Col-0 controls (47.58 ± 2.12 days), *LgFKF1*-OE lines -OE#1 (42.23 ± 1.87 days), -OE#2 (42.16 ± 2.01 days), and -OE#3 (42.37 ± 1.79 days) flowered approximately 5 days earlier, with significantly fewer rosette leaves at flowering (Fig. 2D and F). Additionally, we observed substantial upregulation of flowering-related genes, including *AtFT* and *AtRGL2*, in the *LgFKF1*-OE transgenic lines compared to the Col-0 controls under both LD and SD conditions (Fig. 2G–H). These results suggested that *LgFKF1* overexpression in *A. thaliana* accelerated flowering and affected the expression of flowering-related genes.

To further confirm the role of *LgFKF1* in flowering in *L. gratissima*, *LgFKF1* was silenced using a TRV-based-VIGS system. At 42 days following TRV infiltration, a substantial decrease of *LgFKF1* in the *LgFKF1*-silenced (*LgFKF1*-VIGS) *L. gratissima* plants was observed compared with the WT controls (Fig. 3A and B). The floral bud differentiation in *LgFKF1*-VIGS plants (46.99 ± 2.45 days) occurred approximately 1 day earlier than in the WT controls (48.45 ± 1.21 days) (Fig. 3C and E). Moreover, compared with the WT controls (87.00 ± 2.38 days), *LgFKF1*-VIGS plants (83.38 ± 1.72 days) flowered approximately 4 days earlier (Fig. 3D and F).

Interestingly, the flowering duration of *LgFKF1*-VIGS plants (12.70 ± 2.78 days) was extended significantly (by approximately 3 days) compared to that in the WT controls (9.93 ± 2.14 days) (Fig. 3G). Additionally, *L. gratissima* plants with simultaneous silencing of *LgFKF1* and *LgPRR7* (*LgFKF1*&*LgPRR7*-VIGS, 13.90 ± 3.24 days) exhibited an approximately 4-day extension in flowering duration compared to the WT controls (9.93 ± 2.14 days) (Fig. 3G). The simultaneous silencing of both *LgPRR7* and *LgFKF1* in *L. gratissima* plants led to a synergistic effect (i.e. “1 + 1 > 2”) on floral bud differentiation in *L. gratissima* plants. More specifically, the floral bud differentiation in *LgFKF1*&*LgPRR7*-VIGS plants (43.85 ± 1.09 days) occurred approximately 5 days earlier than in the WT controls (48.45 ± 1.21 days) (Fig. 3C and E), which was in turn earlier than in *LgPRR7*-VIGS (45.56 ± 2.70 days) and *LgFKF1*-VIGS (46.99 ± 2.45 days) plants (Fig. 3E). However, the effect of simultaneous silencing of both *LgPRR7* and *LgFKF1* on flowering time did not conform to this observed pattern. More specifically, *LgFKF1*&*LgPRR7*-VIGS plants flowered at 84.31 ± 2.11 days (Fig. 3F), approximately 3 days earlier compared to the WT controls (87.00 ± 2.38 days), but later than the *LgPRR7*-VIGS (81.56 ± 1.42 days) and *LgFKF1*-VIGS (83.38 ± 1.72 days) plants (Fig. 3F). The synergistic effect of *LgPRR7* and *LgFKF1* silencing on floral bud differentiation (Fig. 3E) but not on flowering time (Fig. 3F) suggests stage-specific roles for the LgPRR7–LgFKF1 module. Floral bud differentiation (an early developmental event) may rely more heavily on the combined effect by both genes, whereas later flowering time regulation might involve compensatory mechanisms or additional factors. This divergence highlights the dynamic nature of flowering regulation, where distinct genetic networks operate at different developmental phases. Additionally, compared with the WT controls, *LgPRR7*-VIGS *L. gratissima* plants showed a significant decrease in *LgFKF1* transcript levels (Fig. 3A and B), *LgFKF1*-VIGS plants exhibited a significant decrease in the levels of *LgPRR7* transcripts (Fig. 3A and B), and *LgFKF1&LgPRR7*-VIGS plants showed significant decreases in the expression of both *LgPRR7* and *LgFKF1* (Fig. 3A and B). These results indicate that *LgPRR7* and *LgFKF1* have a synergistic effect in the regulation of floral bud differentiation in *L. gratissima*.

To determine whether *LgFKF1* silencing affects the expression of downstream flowering-related genes, we analyzed the expression levels of flowering-related genes in *LgFKF1*-VIGS *L. gratissima* plants. Compared with the WT controls, *LgFKF1*-VIGS *L. gratissima* plants showed substantially higher levels of *LgFT* and *LgRGL2* transcripts in the leaves (Fig. 3H), and of *LgLFY* and *LgAP1* transcripts in the buds or the flowers (Fig. 3I), but significantly lower levels of *LgCOL1*2 transcript in the leaves (Fig. 3H), at 42 d after TRV infiltration.

### LgFKF1 interacts with LgRGL2 and LgCOL12

**Figure 5:**
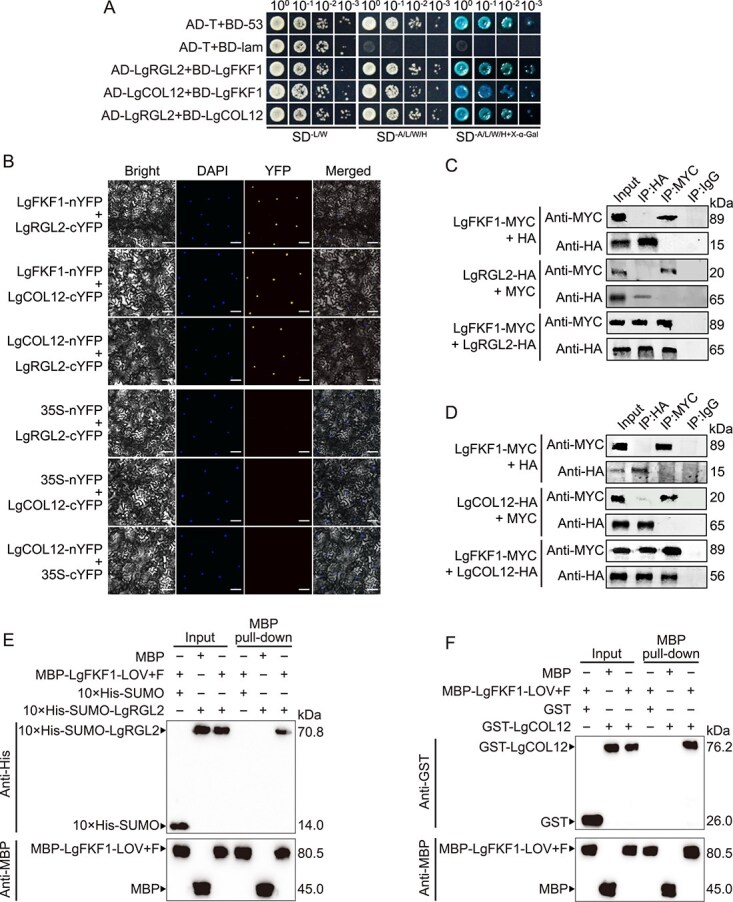
Pairwise interactions among LgFKF1, LgCOL12, and LgRGL2. **(A)** Yeast two-hybrid assays showing pairwise interactions among LgFKF1, LgCOL12, and LgRGL2 in yeast. Yeast was grown on synthetic double dropout (−L/W), quadruple dropout (−A/L/W/H), or quadruple dropout supplemented with X-α-Gal (−A/L/W/H + X-α-Gal) medium. A (Adenine), L (Leucine), W (Tryptophan), H (Histidine). BD, bait protein fused to GAL4 DNA-binding domain; AD, prey protein fused to GAL4 activation domain. The combinations pGADT7-T + pGBKT7–53 and pGADT7-T + pGBKT7-lam were used as the positive and negative controls, respectively. **(B)** Bimolecular fluorescence complementation (BiFC) assays showing pairwise interactions among LgFKF1, LgCOL12, and LgRGL2 in tobacco leaves. The nuclei are indicated by 4,6-diamidino-2-phenylindole dihydrochloride (DAPI) staining. Scale bar: 25 μm. **(C)** Co-immunoprecipitation (Co-IP) assay showing the interaction of LgFKF1 and LgRGL2 in tobacco leaves. Protein samples were extracted from tobacco leaves transiently expressing LgFKF1-MYC and LgRGL2-HA. Immunoprecipitates obtained using anti-MYC or anti-HA magnetic microbeads were analyzed using immunoblotting with anti-MYC and anti-HA antibodies, respectively. Input (crude extracts) and IgG antibody were used as positive and negative controls, respectively. **(D)** Co-IP assay showing the interaction between LgFKF1 and LgCOL12 in tobacco leaves. Protein samples were extracted from tobacco leaves transiently expressing LgFKF1-MYC and LgCOL12-HA. Immunoprecipitates obtained with anti-MYC or anti-HA magnetic microbeads were analyzed using immunoblotting with anti-MYC and anti-HA antibodies, respectively. Input (crude extracts) and IgG antibody were used as positive and negative controls, respectively. **(E)***In vitro* pull-down assay showing the direct interaction between LgFKF1 and LgRGL2. LgFKF1-LOV + F represents the LgFKF1 N-terminal LOV and F-box domains. Recombinant 10 × His-SUMO-LgRGL2 was incubated with immunoprecipitated MBP-LgFKF1-LOV + F. Proteins were detected using immunoblotting with anti-His or anti-MBP antibodies. **(F)***In vitro* pull-down assay showing the direct interaction between LgFKF1 and LgCOL12. Recombinant GST-LgCOL12 was incubated with immunoprecipitated MBP-LgFKF1-LOV + F. Proteins were detected using immunoblotting with anti-GST or anti-MBP antibodies. The “+” or “−” in (**E** and **F**) represent the presence or absence of proteins, respectively.

While investigating the effect of altering *LgPRR7* or *LgFKF1* expression on downstream genes in *L. gratissima*, we observed that *LgPRR7* and *LgFKF1* individually or synergistically affected the expression of the photoperiod pathway transcription regulator gene *LgCOL12* and the GA signaling transcription regulator gene *LgRGL2* (Fig. 3H). These findings prompted us to assess potential interactions between LgFKF1 or LgPRR7 and LgCOL12 or LgRGL2. Yeast two-hybrid assays revealed interactions between LgFKF1 and LgCOL12, LgFKF1 and LgRGL2, as well as LgCOL12 and LgRGL2 (Fig. 5A), whereas LgPRR7 did not interact with either LgCOL12 or LgRGL2 (Fig. S11A). Pairwise interactions *in planta* among LgFKF1, LgCOL12, and LgRGL2 were further confirmed using BiFC and Co-IP assays (Fig. 5B–D). More specifically, the BiFC results revealed strong reconstituted yellow fluorescent protein signals in the nuclei of tobacco leaf cells co-expressing LgFKF1-nYFP and LgRGL2-cYFP or LgCOL12-cYFP, as well as LgRGL2-cYFP and LgCOL12-nYFP (Fig. 5B). Co-IP results showed that LgFKF1-MYC was precipitated by LgRGL2-HA or LgCOL12-HA (Fig. 5C and D) and that LgCOL12-HA was precipitated by LgRGL2-MYC (Fig. S11B). We further confirmed direct interactions among LgFKF1, LgRGL2, and LgCOL12 in *in vitro* pull-down assays (Fig. 5E and F; Fig. S11C), and found that the N-terminal LOV and F-box domains of LgFKF1 mediated the interactions with LgRGL2 or LgCOL12 (Fig. 5E and F). These findings indicate physical interactions among LgFKF1, LgCOL12, and LgRGL2 *in vivo* and *in vitro*, suggesting that they are potentially part of the same protein complex.

## Discussion

### *LgPRR7* or *LgFKF1* orthologs play opposite roles in flowering-time regulation in short-day and long-day plants, and exhibit flowering convergence in short-day or long-day plants

The photoperiod-dependent flowering pathway is a characteristic circadian-controlled event. As critical components of the clock, *PRR7* and *FKF1* have been reported to play roles in modulating photoperiod flowering pathways in several plants [23–34]. In this study, we confirmed that *LgPRR7* and *LgFKF1* are responsive to the photoperiod and suppress flowering in *L. gratissima* (Figs. 1E and F and 3C–F). Our findings are consistent with those of previous studies on short-day plants, including *O. sativa* and *G. max* [27, 28, 33]. However, orthologs of *LgPRR7* and *LgFKF1* play opposing roles in the long-day plants *A. thaliana* and *Populus* [24, 32, 38]. Specifically, *LgPRR7* and *LgFKF1* orthologs function as flowering inhibitors in short-day plants. In *O. sativa*, *OsPRR73* delays heading under short-day conditions by inhibiting *Ehd1* expression [28]. In *G. max*, *GmPRR37* downregulates *GmFT2a* and *GmFT5a* expression and upregulates expression of *GmFT1a*, thereby delaying flowering under long-day conditions [27]. *GmFKF1a/b* delays flowering in *G. max* under both long-day and short-day conditions via the FKF1s-E1s-FT2a/5a module [33]. In contrast, *LgPRR7* and *LgFKF1* orthologs promote flowering in several long-day plants. *AtPRR7* facilitates *A. thaliana* flowering under long-day conditions by suppressing CDF1-mediated inhibition of *CO* [24]. *AtFKF1* promotes flowering in *A. thaliana* under long-day conditions through the FKF1-COP1-CO regulatory cascade [32]. In *Populus*, *PRR7* and *FKF1* are likely to regulate *FT2* expression to facilitate apical shoot growth under long-day conditions [38, 39]. These different roles can be attributed to genetic structure variations occurring during flowering evolution and domestication [40, 41]. In the present study, we found that the effect of ectopic overexpression of *LgPRR7* and *LgFKF1* on flowering in the long-day plant *A. thaliana* (Fig. 2A–F) were consistent with previous findings [24, 32], implying the conservation of *PRR7* and *FKF1* orthologs*.* Conversely, our studies revealed that *LgPRR7* and *LgFKF1* repressed flowering in the short-day plant *L. gratissima* by affecting the expression of the floral activators *LgFT*, *LgLFY*, and *LgAP1* (Fig. 3A–F and H-I).

### Silencing of *LgPRR7* and *LgFKF1* prolongs flowering duration in *L. gratissima*

For ornamental plants, extended flowering duration is particularly valuable. Flowering duration is influenced by various factors. Observational data from subalpine meadow shrubs in Colorado revealed that early-flowering species exhibited extended flowering duration when exposed to premature snowmelt or elevated spring soil temperatures [42]. Moreover, field experiments have demonstrated that the application of nitrogen fertilizer prolonged flowering duration in rice [43]. The *Photoperiod-D1a* (*Ppd-D1a*) allele of wheat, a member of the *PRR* gene family [44], has been found to accelerate initiation of flowering and to prolong the flowering duration in wheat significantly [45]. Under natural conditions, the flowering duration of wild-type *L. gratissima* inflorescences is 6–10 days. In this study, the early-flowering plants (*LgPRR7*-VIGS, *LgFKF1*-VIGS, and *LgFKF1*&*LgPRR7*-VIGS) exhibited extended flowering duration, representing a significant prolongation of 2–4 days compared to the controls (Fig. 3G). This phenotype may be associated with *LgPRR7* [45]. While the precise molecular mechanisms underlying this phenomenon require further investigation, our findings strongly indicate that *LgPRR7* and *LgFKF1* function as multifunctional regulators in the *L. gratissima* flowering process. A prolonged flowering duration will extend the sales period for cut flowers and potted plants of *L. gratissima*, and will meet promotional needs during long holidays such as Spring Festival or Christmas, significantly enhancing the efficiency of horticultural production and market competitiveness for this ornamental species. Furthermore, this discovery provides scientific insights into the regulation of flowering time and duration which may have application in other ornamental flowers.

### GA mediates the *LgPRR7*- and *LgFKF1*-regulated photoperiodic flowering in *L. gratissima* through the LgRGL2 protein

GA signaling plays a regulatory role in flowering in *L. gratissima*. In this study, we found that GA signaling was involved in the *LgPRR7*- and *LgFKF1*-regulated flowering via *LgRGL2*-encoded DELLA protein in *L. gratissima* (Figs 3H and 5)*.* In *A. thaliana*, FKF1 physically interacts with RGA and GAI (DELLA proteins) both *in vitro* and *in vivo* to regulate flowering, but not with RGL2 [46]. In contrast, in this study, we found that LgRGL2 interacted with LgFKF1 both *in vitro* and *in vivo* (Fig. 5) and that *LgRGL2* expression increased in *LgFKF1*-VIGS *L. gratissima* plants at 42 days after TRV infiltration (Fig. 3H), indicating that LgFKF1 may negatively regulate *LgRGL2* expression. Interestingly, we found that *LgPRR7* silencing also increased *LgRGL2* expression at 42 days after TRV infiltration (Fig. 3H), although LgPRR7 did not interact with LgRGL2 (Fig. S11A). This implies that LgPRR7 may indirectly affect *LgRGL2*. GA_4_ is considered the most crucial bioactive GA in regulating plant flowering time [47]. However, our study showed that levels of bioactive GA_7_ were more different between the SH and LH treatments than were levels of the other bioactive GAs, including GA_1_, GA_3_, and GA_4_ (Fig. S2A). Moreover, compared to GA_3_, GA_7_ showed a higher degree of correlation with the GA receptor gene *GID1B* and the GA signal suppressor gene *RGL2* (Fig. S2C; Table S1). We speculate that GA_7_, as one of the key bioactive GAs regulating flowering in *L. gratissima*, mediates *LgPRR7*- and *LgFKF1*-regulated photoperiodic flowering via the LgRGL2 protein.

### The clock LgPRR7–LgFKF1 module negatively regulates flowering in *L. gratissima*

The clock-dependent photoperiodic flowering pathway involves complex crosstalk between regulatory networks. In this study, we revealed that the clock LgPRR7–LgFKF1 module suppressed flowering in *L. gratissima*. Previous studies in *A. thaliana* have indicated that FKF1 fine-tuned the pace and robustness of circadian oscillations by interacting with PRR5 and TOC1 [48]. However, whether FKF1 interacts with PRR7 to regulate flowering time has not yet been reported. Our study revealed that LgPRR7 interacted with LgFKF1 (Fig. 4), and that silencing these genes individually or together downregulated the expression of the other gene (Fig. 3A and B). Simultaneously silencing both genes synergistically promoted *L. gratissima* flower bud differentiation (Fig. 3C and E), implying that *LgPRR7* and *LgFKF1* regulate *L. gratissima* flower bud differentiation by forming the LgPRR7–LgFKF1 module. The reciprocal regulation between *LgPRR7* and *LgFKF1* at both transcriptional and protein levels (Figs 3A and 4) may constitute a positive feedback loop to amplify photoperiodic signals. For example, *LgPRR7* silencing led to the down-regulation of *LgFKF1* transcription (Fig. 3A), whereas the two protein interactions (Fig. 4) may further stabilize the complex. Such a mechanism could enhance the robustness of circadian output in short-day plants, where precise timing of floral transition is critical for survival [1]. Analogous regulatory loops, such as the CCA1-LHY-TOC1 circuit in Arabidopsis [49], are known to stabilize circadian rhythms under fluctuating environmental conditions. Additionally, the LgPRR7–LgFKF1 module may act as a transcriptional repressor complex that directly or indirectly suppresses flowering-related genes. The interaction between LgPRR7 and LgFKF1 is likely to stabilize the levels of their proteins or enhance their capacity to bind to the regulatory elements of target genes. For instance, the CCT domain of LgPRR7, known for DNA-binding activity in other PRR proteins [50, 51], may recruit LgFKF1 to form a transcriptional repressor complex. Future studies will focus on chromatin immunoprecipitation (ChIP) assays to identify the direct targets of the LgPRR7–LgFKF1 complex.

In *A. thaliana*, the CO protein induces *FT* expression to promote flowering, while FKF1 inhibits COP1 homodimerization via direct interaction, thereby suppressing COP1-mediated CO degradation [32]. DELLA proteins interact directly with CO to inhibit activation of its transcription [52]. COL12, a member of the CO family [53], functions differently from CO. COL12 inhibits CO activity via direct interaction, resulting in delayed flowering [54]. Interactions among COL12, FKF1, and RGL2 have not yet been reported. Our study identified interactions between LgCOL12 and both LgFKF1 and LgRGL2, as well as between LgFKF1 and LgRGL2 (Fig. 5; Fig. S11B and C), implying that LgFKF1, LgCOL12, and LgRGL2 possibly form an LgFKF1–LgCOL12–LgRGL2 complex to regulate flowering in *L. gratissima*. The LgPRR7–LgFKF1 interaction is likely to serve as a regulatory hub, integrating circadian timing with photoperiodic signals, while the LgFKF1–LgCOL12–LgRGL2 complex putatively bridges the circadian and GA pathways. Mechanistically, LgFKF1 may act as a scaffold, recruiting LgCOL12 (a photoperiod pathway repressor) and LgRGL2 (a GA signaling repressor) to form a multiprotein complex that synergistically inhibits flowering. We speculate that the complex may suppress *LgFT* expression via two mechanisms: (i) LgCOL12 inhibition of CO-mediated transcriptional activation of *LgFT* [55] and (ii) LgRGL2 blocking GA-mediated activation of floral integrators [56]. The physical interaction between these proteins ensures coordinated repression, enabling *L. gratissima* to fine-tune flowering in response to both external and internal cues.

**Figure 6:**
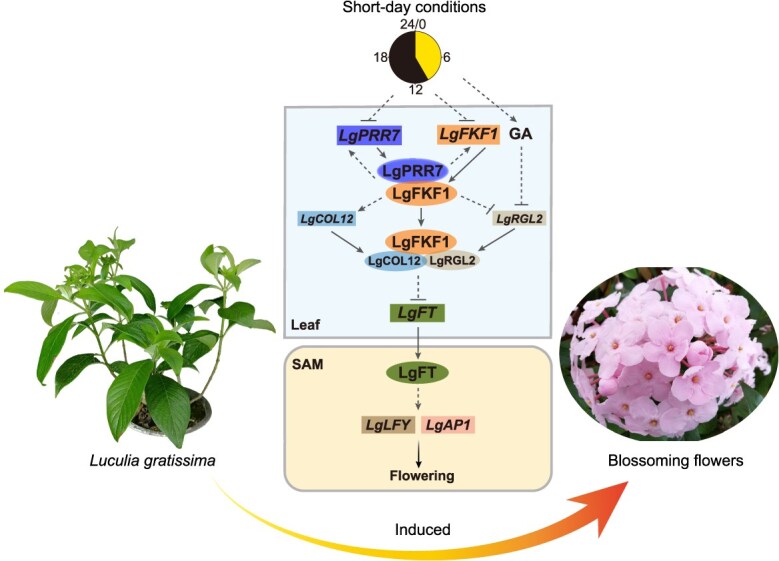
Possible working model depicting how the clock module LgPRR7-LgFKF1 may regulate flowering in *Luculia gratissima*. The expression of *LgPRR7* and *LgFKF1* is controlled by the photoperiod, and the LgPRR7–LgFKF1 protein complex reinforces its own stability via a positive feedback loop that amplifies *LgPRR7* and *LgFKF1* expression. The LgPRR7-LgFKF1 complex indirectly activates *LgCOL12* while repressing *LgRGL2* expression. The LgFKF1 protein further recruits LgCOL12 and LgRGL2 to form the LgFKF1–LgCOL12–LgRGL2 complex, which negatively regulates the expression of *LgFT*, *LgLFY*, and *LgAP1*, thereby fine-tuning flowering in *L. gratissima*. In addition, GA is possibly involved in this process through *LgRGL2*. Squares and circles indicate genes and proteins, respectively. The arrows and T-shaped bars represent positive and negative regulation, respectively. The solid and dashed lines indicate direct and hypothetical/indirect relationships between the two points, respectively.

Based on previous findings and our current results, we propose a working model to illustrate how the LgPRR7–LgFKF1 module controls *L. gratissima* flowering (Fig. 6). Briefly, the expression of both *LgPRR7* and *LgFKF1* is controlled by the photoperiod pathway [24, 29]; the proteins encoded by these genes interact physically to form the LgPRR7–LgFKF1 complex, which reinforces its own stability via a positive feedback loop. The LgPRR7–LgFKF1 complex indirectly promotes *LgCOL12* while repressing *LgRGL2* expression. Moreover, LgFKF1 recruits LgCOL12 and LgRGL2 to form the LgFKF1–LgCOL12–LgRGL2 complex. The LgPRR7–LgFKF1 module, via the LgFKF1–LgCOL12–LgRGL2 complex, indirectly inhibits the expression of downstream flowering-related genes, including *LgFT*, *LgLFY*, and *LgAP1* [3, 54], thereby fine-tuning flowering in *L. gratissima*. Additionally, endogenous bioactive GA (possibly GA_7_) may participate in this process by negatively regulating *LgRGL2*. Nonetheless, further studies are required to clarify how the LgPRR7–LgFKF1 and LgFKF1–LgCOL12–LgRGL2 complexes regulate the downstream flowering-related genes in *L. gratissima*, and whether the application of exogenous GA_7_ affects *L. gratissima* flowering. The LgPRR7–LgFKF1 module provides genetic targets for the genetic improvement of flowering-time in *L. gratissima* through selecting various combinations from the clock-module-regulated pathway, which helps to optimize the photoperiod and planting time.

The role of the LgPRR7–LgFKF1 module as a flowering repressor in the short-day plant *L. gratissima* contrasts sharply with the flowering-promoting functions of its orthologs in long-day plants (e.g. AtPRR7/AtFKF1 in Arabidopsis) [24, 32]. This functional divergence underscores the evolutionary plasticity of circadian clock components in adapting to distinct photoperiodic environments. For instance, while *OsPRR73* in rice (a short-day monocot) delays flowering under long days by repressing *Ehd1* [28], *GmPRR37* in soybean (a short-day dicot) inhibits flowering via *GmFT2a/5a* suppression [27]. Similarly, our discovery of the LgFKF1–LgCOL12–LgRGL2 complex reveals a novel regulatory node integrating circadian and GA signaling, a mechanism that has not yet been reported in other species. These insights advance our understanding of how conserved clock components are repurposed across plant lineages to optimize flowering timing, offering a framework for comparative studies in both long-day and short-day, as well as monocotyledonous and dicotyledonous plants.

## Materials and methods

### Plant materials and growth conditions

One-year-old cuttings of *L. gratissima* ‘Xiangfei’ from the same three-year-old mother plant were obtained as described previously [37] from the central Yunnan Plateau experimental station of the Institute of Highland Forest Science, CAF (25°13’ N, 102°12′ E; 1826 m a.s.l.). As previously described [37], to prevent short-day induction, rooted cuttings with apical buds removed were maintained in a natural light greenhouse with additional supplemental light from 22:00 to 2:00 (night-break treatment) until initiation of subsequent different photoperiod treatments. Cuttings with the same number of branches and 5–6 stem nodes per branch were subjected to either a controlled short-day (SH, 10-hour light from 8:00 to 18:00 /14-hour dark at 20°C) or a controlled long-day (LH, 12-hour light from 8:00 to 20:00/12-hour dark with a 4-hour night break from 22:00 to 2:00 at 20°C) in culture chambers with an LED cool white light intensity of 300 μmol·m^−2^·s^−1^ and 60% relative humidity. Shoot apices and mature leaves were collected from the main branches of healthy SH and LH plants between 10:00 and 10:30 a.m. at 10, 20, and 30 days after initiation of photoperiod treatment. For each stage, 10 shoot apices and two mature leaves were packed together into each of the eight biological replicates. Of these, one was fixed immediately in a 50% FAA solution for morphoanatomical analysis, one was used for gene cloning, and the remaining six were quick-frozen in liquid nitrogen and stored at −80°C for GA content determination and RNA extraction.

### Morphoanatomical observation and measurement of endogenous GA content

FAA-fixed stem apices were sectioned and examined using light microscopy as previously described [37]. Eighteen endogenous GAs (GA_1/3/4/5/6/7/8/9/13/14/15/19/20/24/29/44/51/53_) were quantified using high-performance liquid chromatography with tandem mass spectrometry (HPLC–MS/MS) (Aglient1290, Nanjing, China; AB Sciex QTRAP 6500+, Nanjing, China) as reported previously [57]. Three biological replicates were measured for each period.

### RNA-seq, data analysis, and RT-qPCR

Total RNA was extracted from mixed samples comprising shoot apices and leaves from SH or LH plants at three stages (10 days [SH10 or LH10], 20 days [SH20 or LH20], and 30 days [SH30 or LH30] after the onset of the photoperiod treatment) for three biological replicates. RNA extraction, cDNA library construction, RNA-seq, *de novo* assembly, gene annotation, and gene function classification were performed as previously described [37]. The expression levels of unigenes derived from the *de novo* assembly were evaluated using the reads per kilobase per million reads (RPKM) method [58]. DEGs between SH and LH samples were identified using DESeq2 software [59], with an FDR < 0.05 and absolute fold change of 1.3 as thresholds. KOBAS version 3.0 [60] was used to identify the significantly enriched Kyoto Encyclopedia of Genes and Genomes (KEGG) metabolic pathways (*P* < 0.05).

RT-qPCR was performed to validate the expression levels of flowering-related genes from the RNA-seq data according to previous procedures [37]. Primer Premier v5.0 software [61] was used to design specific primers for each gene (Table S5). RNA extraction, reverse transcription, and RT-qPCR were performed as described previously [37]. The relative expression levels of genes were calculated using the 2^−ΔΔ*C*t^ method [62]. Gene expression was normalized using the geometric mean of three previously reported internal reference genes, *LgActin*, *LgEF-1α*, and *LgTUB* [63, 64].

### Co-expression network construction

Co-expression network modules were generated based on 19 943 unigenes (all samples with mean RPKM ≥1 and coefficient of variation [CV] > 0.1) using the WGCNA R package [65]. The parameters were set as follows: soft threshold power = 6, minModuleSize = 100, and mergeCutHeight = 0.3, with the remaining parameters set to the default settings. The flowering-related proteins identified from the WGCNA modules were mapped to the *A. thaliana* protein sequences. A PPI network was constructed using STRING version 11.5 [66]. Module eigengene values were calculated to test their associations with GAs. Through module–trait correlation analysis, genes related to GA biosynthesis and signal transduction were identified, and the interaction pairs between the genes and GAs with |cor| ≥ 0.5 were used to construct a GA regulatory network. Network visualization was performed using Cytoscape software version 3.9.1 [67].

### Gene cloning and sequence analysis

Total RNA was extracted by mixing all the shoot apices and leaf samples using a Trizol reagent kit (Invitrogen, Carlsbad, CA, USA) and reverse-transcribed using a RevertAid™ First Strand cDNA Synthesis Kit (Thermo Fisher Scientific, Waltham, MA, USA) according to the respective manufacturer’s protocols. Based on the sequence of the *LgPRR7* (Lg44686) cDNA fragment obtained from the transcriptome in *L. gratissima*, we performed rapid amplification of cDNA ends (RACE) as previously described [68] to clone the full-length cDNA sequence of *LgPRR7* gene. The *LgFKF1* gene sequence was obtained from our previous study [69]. The full-length coding sequences (CDS) of *LgCOL12* and *LgRGL2* were cloned using the KOD FX Neo Kit (Toyobo, Osaka, Japan) based on the sequences of *LgCOL12* (Lg34796) and *LgRGL2* (Lg39096) cDNA fragments, respectively, which were obtained from the transcriptome of *L. gratissima*, following the manufacturer’s protocol. Genomic DNA (gDNA) was extracted from the samples by mixing all shoot apices and leaves using a Genomic DNA Extraction Kit (Aidlab, Beijing, China). Gene-specific primers were designed to amplify gene fragments and confirm the absence of introns based on the full-length cDNA sequences of *LgPRR7* and *LgFKF1*, with the accurately sequenced fragments used for subsequent 5′ Walking tests. According to the protocol of the Genome Walking Kit (Takara, Dalian, China), two rounds of nested PCR were performed for each of two 5′ Walking tests. The promoter sequences of *LgPRR7* and *LgFKF1* were cloned based on the 5′ Walking test results. All primers (Table S5) were designed using Primer Premier v5.0 software [61].

The cDNA sequences of *LgPRR7* and *LgFKF1* were translated into protein sequences using ORF Finder (https://www.ncbi.nlm.nih.gov/orffinder/). The subcellular localization of LgPRR7 and LgFKF1 was predicted using the Plant-mPLoc platform (http://www.csbio.sjtu.edu.cn/bioinf/plant-multi/). Conserved protein domains were predicted using CD-Search (https://www.ncbi.nlm.nih.gov/Structure/cdd/wrpsb.cgi). Multiple homologous protein sequences downloaded from the NCBI database were aligned using Clustal Omega (https://www.ebi.ac.uk/Tools/msa/clustalo/), and the results were visualized using JALVIEW [70]. MEGA-X software [71] was used to align the homologous protein sequences of LgPRR7 and LgFKF1 and construct phylogenetic trees based on the neighbor-joining method (1000 bootstrap replicates). The motifs of the PRR7 and FKF1 proteins across different species were identified using MEME (http://meme-suite.org/tools/meme). TBtools software [72] was used to visualize the generated phylogenetic trees and motif results.

The transcription start sites (TSSs) of the *LgPRR7* and *LgFKF1* promoters were predicted using BDGP (https://www.fruitfly.org/seq_tools/promoter.html). PlantCARE (http://bioinformatics.psb.ugent.be/webtools/plantcare/html/) was used to predict the *cis*-acting elements (CAEs) in the *LgPRR7* and *LgFKF1* promoters. The promoter sequences of *LgPRR7* and *LgFKF1* were uploaded to the Plant ChIP-seq database of PlantPAN 3.0 (http://pcbase.itps.ncku.edu.tw) for ChIP-seq data retrieval and to predict transcription factor binding sites (TFBSs) and their corresponding transcription factors (TFs). The predicted CAEs on the promoters, TFBSs, and corresponding TFs were visualized using TBtools software [72].

### Spatiotemporal and diurnal expression analysis of *LgPRR7* and *LgFKF1*

As *L. gratissima* is a typical obligate short-day plant [36], and the floral bud differentiation was observed only under SH conditions in this study, we focused on analyzing the expression patterns of *LgPRR7* and *LgFKF1* transcripts during flowering exclusively under SH treatment. To comprehensively characterize the expression patterns of *LgPRR7* and *LgFKF1*, we conducted spatiotemporal and diurnal transcriptional profiling in *L. gratissima* under SH conditions. For spatiotemporal expression analysis, root, stem, leaf, and bud tissues were harvested between 10:00 and 10:30 a.m. at 10, 20, and 30 days following initiation of SH treatment. To investigate the diurnal rhythms, further leaf samples were collected at 4-hour intervals over a 72-hour period (spanning three consecutive 10-hour light/14-hour dark cycles) starting from 30 days after the onset of SH treatment. All experiments were performed under SH conditions (10-hour light/14-hour dark photoperiod, light intensity: 300 μmol·m^−2^·s^−1^, temperature: 20°C, relative humidity: 60%). Total RNA was extracted from tissues, and relative transcript levels of *LgPRR7* and *LgFKF1* were quantified using RT-qPCR. The previously validated reference genes *LgActin*, *LgEF-1α*, and *LgTUB* [63, 64] were employed for data normalization. RT-qPCR analysis followed the same protocol as described previously [37], and three biological replicates were analyzed per time point and tissue. The primer sequences are listed in Table S5.

### Subcellular localization of LgPRR7 and LgFKF1 proteins

Full-length CDSs of *LgPRR7* or *LgFKF1* without termination codons were cloned into pAN580-35S-*GFP* vectors to generate the fusion vectors 35S::*LgPRR7*-*GFP* and 35S::*LgFKF1*-*GFP*, respectively. The 35S::*Ghd7*-*CFP* (nuclear marker) plasmid was then co-transformed into *A. thaliana* protoplasts with 35S::*GFP*, 35S::*LgPRR7*-*GFP*, or 35S::*LgFKF1*-*GFP* plasmids, as previously described [73]. After incubation at 25°C in low light for 8–10 h, GFP and CFP fluorescence was observed using a laser confocal scanning microscope (Olympus, Tokyo, Japan).

### *A. thaliana* transformation

The full-length cDNA sequences of *LgPRR7* and *LgFKF1* were cloned into the pCAMBIA1300S vector to generate the recombinant overexpression plasmids 35S::*LgPRR7* (OE) and 35S::*LgFKF1* (OE), respectively. Then, the pCAMBIA1300S empty vector (EV) and recombinant overexpression plasmids were separately transformed into *Agrobacterium tumefaciens* GV3101 to transform the *A. thaliana* ecotype Columbia-0 (Col-0) via the floral-dipping method [74]. Independent transgenic *A. thaliana* lines were screened on 1/2 MS medium supplemented with 51.6 mM hygromycin (Solarbio, Beijing, China). The putative transformants were confirmed via a PCR amplification. The *T*_4_ generation transgenic lines were used for further analysis. Col-0 and transgenic *A. thaliana* plants were subjected to either a controlled long-day (LD, 16-hour light from 6:00 to 22:00 /8-hour dark) or a controlled short-day (SD, 8-hour light from 8:00 to 16:00/16-hour dark) in growth chambers at 22°C with an LED white light intensity of 300 μmol·m^−2^·s^−1^ and 60% relative humidity.

### VIGS assay

To silence *LgPRR7* and *LgFKF1*, partial CDS fragments of *LgPRR7* (422 bp) and/or *LgFKF1* (405 bp) were cloned into pTRV2 vectors to obtain the pTRV2-*LgPRR7*, pTRV2-*LgFKF1*, pTRV2-*LgFKF1*&*LgPRR7* recombinant plasmids (Fig. S12A). According to previously described protocols [75, 76] with some modifications, *LgPRR7* and/or *LgFKF1* were silenced in one-year-old cuttings of *L. gratissima* with the same number of branches and 5–6 stem nodes per branch. Briefly, cultures of *A. tumefaciens* GV3101 transformed with pTRV1, pTRV2-*LgPRR7*, pTRV2-*LgFKF1*, pTRV2-*LgFKF1*&*LgPRR7*, pTRV2-*NbPDS* (positive control; Fig. S12B), or pTRV2 (negative control) were prepared (OD_600_ = 0.6–1.0), after which the transformed GV3101 cells were resuspended in an infiltration buffer (10 mM MgCl_2_, 10 mM MES, 200 μM acetosyringone; pH 5.6) and adjusted to an OD_600_ of 1.0. Next, pTRV1 and pTRV2 or pTRV2 derivatives were mixed at a 1:1 volume ratio, and the mixtures were kept in the dark at 25°C for 4 h. Before infiltrating the plants, 1‰ (v/v) Silwet L-77 was added to the mixtures. Agro-infiltration was performed with a needleless 1 mL syringe into the abaxial surface of the two leaves, infiltrating the entire leaf. After two weeks of growth in culture chambers at 18°C under LH conditions, the VIGS-treated *L. gratissima* plants and the controls were subjected to SH and LH treatments at 20°C in culture chambers.

### Phenotype observation and analysis of flowering-related gene expression

In the phenotypic analysis of *LgPRR7-* and *LgFKF1-*overexpressing *A. thaliana* transgenic lines (≥ 25 per line), we recorded the number of days from sowing to reaching a bolt length of 1 cm (bolting time), the number of days from sowing to the opening of the first flower (flowering days), and the number of rosette leaves at bolting and flowering. For the *L. gratissima* VIGS plants (≥ 25 per line), we recorded the floral bud differentiation time (the number of days from agro-infiltration to floral bud differentiation), flowering time (the number of days from agro-infiltration to flowering), and flowering duration (the number of days from the opening of the first flower to the wilting of the last flower within an inflorescence, by recording the number of open flowers in each inflorescence daily and calculating the time span between these two events).

To investigate the influence of *LgPRR7* and *LgFKF1* overexpression or silencing on flowering-related gene expression in *A. thaliana* and *L. gratissima*, RT-qPCR analysis was performed according to previous research [37]. For heterologously transformed *A. thaliana* plants, new rosette leaves and buds/flowers under SD or LD treatments were harvested and mixed into each of three biological duplicate samples. *AtEF1α* was used as the internal control for *A. thaliana*. For *L. gratissima* VIGS plants, new leaves and buds/flowers at different stages subjected to SH treatment were collected separately, with three biological replicates per stage and tissue sample. The previously validated reference genes *LgActin*, *LgEF-1α*, and *LgTUB* [63, 64] were used as internal controls for *L. gratissima*. The analyzed genes and their primer sequences are listed in Table S5.

### Yeast two-hybrid assay

The full-length CDSs of *LgFKF1*, *LgCOL12*, and *LgRGL2* were cloned into pGBKT7 vectors (bait), whereas the full-length CDSs of *LgPRR7*, *LgCOL12*, and *LgRGL2* were cloned into the pGADT7 vector (prey). The primers used are listed in Table S5. Following the instructions of the Matchmaker® Gold Yeast Two-Hybrid System (Clontech, Mountain View, CA, USA), the bait and prey constructs were co-transformed with heat-denatured salmon sperm DNA into the yeast strain Y2HGold. Simultaneously, pGBKT7 recombinant vectors were co-transformed with the empty pGADT7 vector into Y2HGold cells to test their self-transcriptional activation activity. Transformed yeast cells were cultured in selective synthetic dropout medium SD/−Leu/−Trp (SD/–LW) plates, and colonies were streaked onto SD/−Ade/−Leu/−Trp/–His (SD/–ALWH) medium and incubated at 30°C. The colonies were further streaked onto SD/−Ade/−Leu/−Trp/–His+X-α-Gal (SD/–ALWH+X-α-Gal) plates and cultured at 30°C for two days to check for possible interactions. Co-transformations with pGADT7-T and pGBKT7–53 or pGBKT7-lam were used as positive and negative controls, respectively.

### BiFC assay

CDSs of *LgFKF1*, *LgCOL12*, and *LgRGL2* without the termination codons were cloned into pSPYNE-MYC vectors, whereas CDSs of *LgPRR7*, *LgCOL12*, and *LgRGL2* without the termination codons were cloned into pSPYCE-HA vectors. The primers used are listed in Table S5. The recombinant plasmids were introduced into *A. tumefaciens* GV3101 cells. Following a previously described protocol [77], the strains corresponding to the positive interaction combination in the yeast two-hybrid assay were used for co-infiltration into four-week-old *Nicotiana benthamiana* (tobacco) leaves. Plants were then grown in an incubator at 25°C under continuous light for 72 h, and fluorescence signals were observed using a laser confocal scanning microscope (Olympus, Tokyo, Japan).

### Co-IP assay

Total protein was extracted from agro-infiltrated tobacco leaves in the BiFC assay using a Plant Protein Extraction Kit (ComWin Biotech, Beijing, China). Co-IP assays were conducted using a Pierce™ Magnetic HA-Tag IP/Co-IP Kit and Pierce™ Magnetic MYC-Tag IP/Co-IP Kit (Thermo Fisher Scientific, Waltham, MA, USA) according to the manufacturer’s instructions. The blots were probed using DyLight 488-conjugated rabbit anti-HA-Tag and Alexa Fluor 647-conjugated mouse anti-MYC-Tag antibodies (Antibodies-Online GmbH, Aachen, Germany), following the manufacturer’s protocol.

### *In vitro* pull-down assay

The two CDS fragments of *LgFKF1* encoding amino acid residues 1–300 (LOV and F-box domains: LgFKF1-LOV + F) and 300–630 (Kelch repeat domains: LgFKF1-Kelch) were independently cloned into the pMAL-c5X-MBP vector; the full-length CDS of *LgRGL2* and the two CDS fragments of *LgPRR7*—encoding residues 97–556 (PR domain: LgPRR7-PR) and 700–790 (CCT domain: LgPRR7-CCT)—were independently cloned into the pET28a-10 × His-SUMO vector; the full-length CDS of *LgCOL12* were cloned into the pGEX-6p-1-GST vector. The primers used are listed in Table S5. All proteins were expressed in *Escherichia coli* BL21 (DE3). The recombinant proteins of MBP, His, and GST were purified using MBPSep Dextrin Agarose Resin, Ni NTA Agarose, and Glutathione Agarose, respectively. The *in vitro* pull-down assay was carried out as previously described [78] with modifications. In brief, 200 μg of the immunoprecipitated MBP-LgFKF1-Kelch was incubated with 200 μg of 10 × His-SUMO-LgPRR7-PR or 10 × His-SUMO-LgPRR7-CCT, 200 μg of the immunoprecipitated MBP-LgFKF1-LOV + F was incubated with 200 μg of 10 × His-SUMO-LgPRR7-PR, 10 × His-SUMO-LgPRR7-CCT, 10 × His-SUMO-LgRGL2, or GST-LgCOL12, and 200 μg of the immunoprecipitated GST-LgCOL12 was incubated with 200 μg of 10 × His-SUMO-LgRGL2, all at 4°C and overnight. The mixture was collected by centrifugation at 500 *g* at 4°C for 2 min, and then washed with phosphate-buffered saline (PBS) buffer three times. Proteins were resolved by SDS-PAGE, transferred to a PVDF membrane, and analyzed using anti-MBP (Proteintech, Wuhan, China), anti-GST (YEASEN, Shanghai, China), or anti-His (Proteintech, Wuhan, China) antibodies.

## Supplementary Material

Web_Material_uhaf110

## Data Availability

The raw data of RNA-seq from this study have been deposited in the NCBI Sequence Read Archive (SRA) database under accession no. PRJNA1109317. The *de novo* transcriptome has been deposited in the NCBI Transcriptome Shotgun Assembly (TSA) database under the accession no. GKUL00000000. The nucleotide sequences generated from this study have been deposited at GenBank under accession numbers MT822705 (the full-length cDNA sequence of *LgPRR7*), MT822704 (the full-length cDNA sequence of *LgFKF1*), PP782638 (the promoter sequence of *LgPRR7*), PP782639 (the promoter sequence of *LgFKF1*), PP782640 (the full-length CDS of *LgCOL12*), and PP782641 (the full-length CDS of *LgRGL2*), respectively.
