# Supplementary material for: The circadian clock module LgPRR7–LgFKF1 negatively regulates flowering time in Luculia gratissima, a woody ornamental plant
Source: Hortic Res. 2025 Apr 24;12(7):uhaf110. doi: 10.1093/hr/uhaf110 (PMC12227992; doi:10.1093/hr/uhaf110)
Supplement: Web_Material_uhaf110 [file web_material_uhaf110.zip › Supplementary_Figures.pdf]

Supplementary Figures

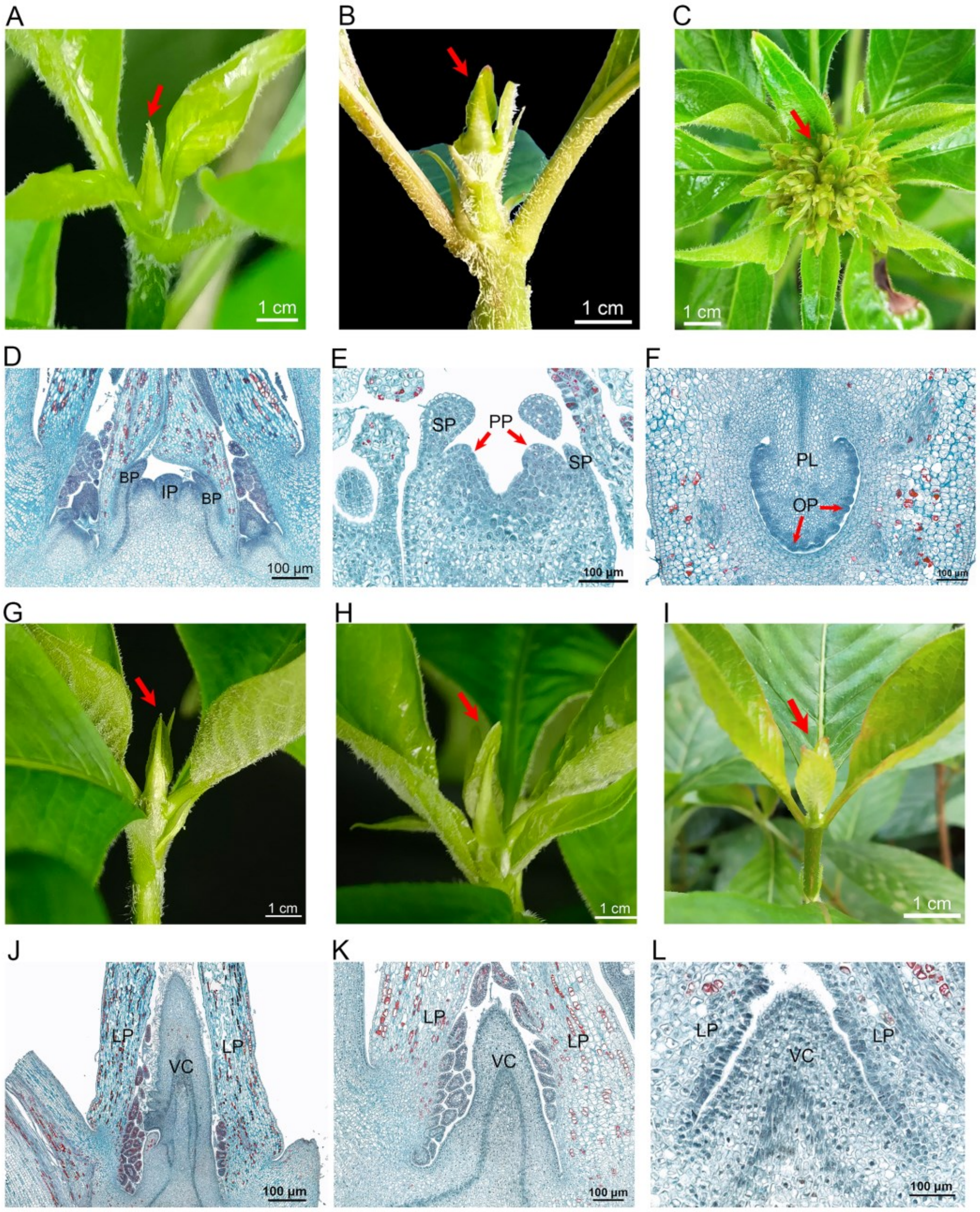

Figure S1. Morphological characteristics of *Luculia gratissima* shoot apices at three stages under different photoperiod treatments. **(A–F)** External morphological **(A–C)** and morphoanatomical **(D–F)** characteristics of *L. gratissima* shoot apices under SH conditions. The development stages of *L. gratissima* shoot apices at 10 **(A, D)**, 20 **(B, E)**, and 30 days **(C, F)** after the onset of SH treatment correspond to the differentiation of the inflorescence primordia, perianth primordia, and pistil primordia, respectively. SH: controlled short-day (10-hour light from 8:00 to 18:00 /14-hour dark at 20 °C). Scale bar in **(A–C)**: 1cm; Scale bar in **(D–F)**: 100 μm. **(G–L)** External morphological **(G–I)** and morphoanatomical **(J–L)** characteristics of *L. gratissima* shoot apices under LH conditions. *L. gratissima* shoot apices at 10 **(G, J)**, 20 **(H, K)**, and 30 days **(I, L)** after the onset of LH treatment remained in the vegetative phase. LH: controlled long-day (12-hour light from 8:00 to 20:00 /12-hour dark with a 4-hour night break from 22:00 to 2:00 at 20 °C). Scale bar in **(G–I)**: 1cm; Scale bar in **(J–L)**: 100 μm. BP: bract primordia; IP: inflorescence primordia; LP: leaf primordia; OP: ovule primordia; PL: placenta; PP: petal primordia; SP: sepal primordia; VC: vegetative cone.

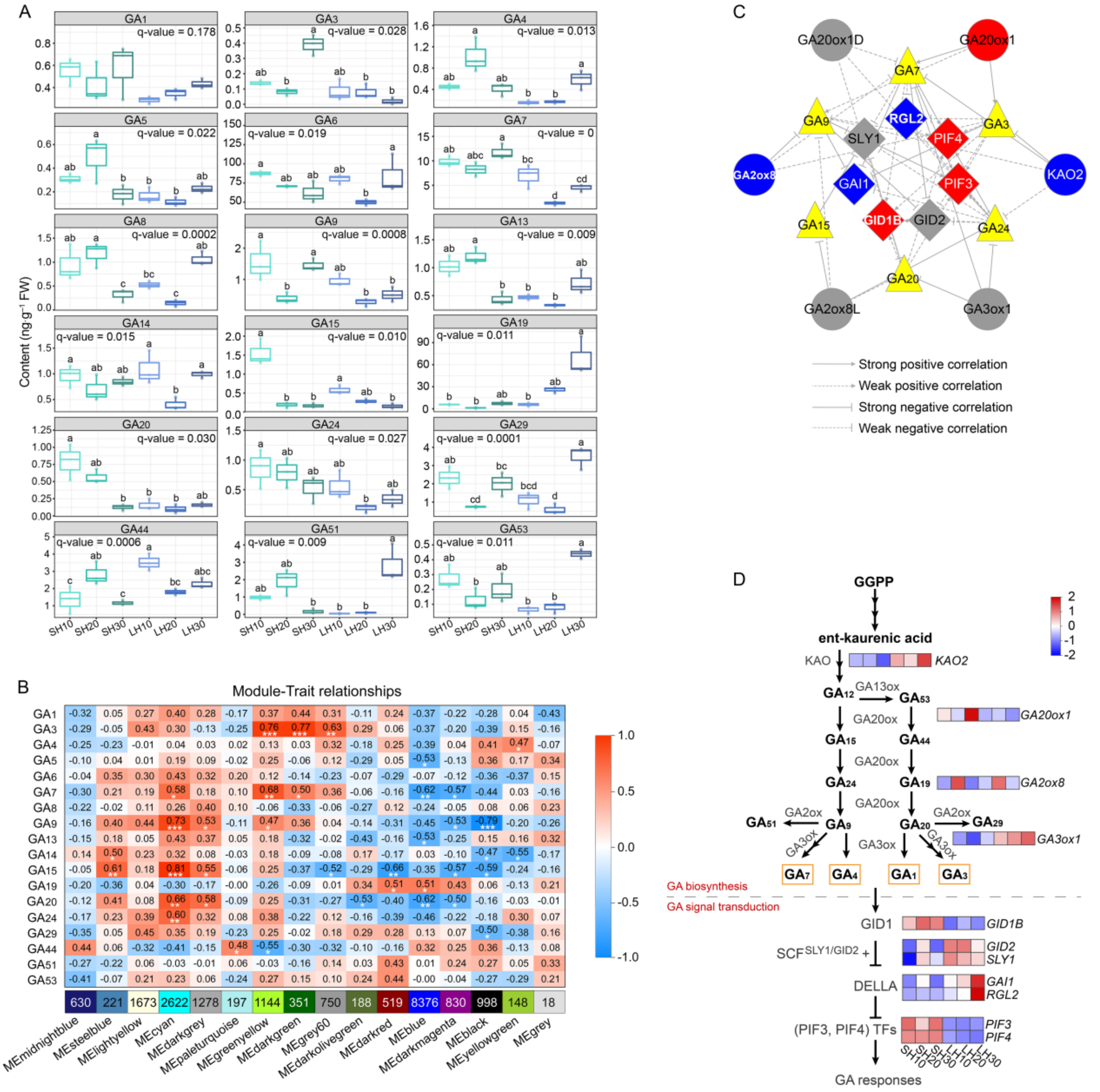

Figure S2. Dynamic changes in the content of 18 endogenous gibberellins (GAs) and analysis of the correlation between GAs and the transcriptome in *Luculia gratissima* grown under different photoperiod treatments. **(A)** Endogenous GA content at three stages in the *L. gratissima* shoot apices and leaves after SH and LH treatments. SH: controlled short-day (10-hour light from 8:00 to 18:00 /14-hour dark at 20 °C). LH: controlled long-day condition (12-hour light from 8:00 to 20:00 /12-hour dark with a 4-hour night break from 22:00 to 2:00 at 20 °C). Data are from three biological replicates ($n = 3$). The *P*-values were adjusted using the False Discovery Rate (FDR), with the adjusted *P*-values represented as q-values. Different lowercase letters indicate significant differences at q-value < 0.05 (Tukey's test). **(B)** Heatmap showing module-GA correlations. The red and blue colors indicate positive and negative correlations, respectively. $*P < 0.05$, $**P < 0.01$, $***P < 0.001$ (Student's *t*-test). **(C)** Correlation network of GA biosynthesis and signal transduction. Gray, red, and blue nodes represent non-differentially expressed, upregulated, and downregulated genes, respectively. Solid and dotted edges represent strong correlations (|cor| ≥ 0.7) and weak correlations (|cor| < 0.7), respectively. The arrow and T-shaped bar edges represent positive (cor > 0) and negative regulation (cor < 0), respectively. **(D)** The expression profile of genes involved in GA biosynthesis and signal transduction pathways. The Z-score normalized RPKM value of genes is represented in a blue (low expression) to red (high expression) scale.

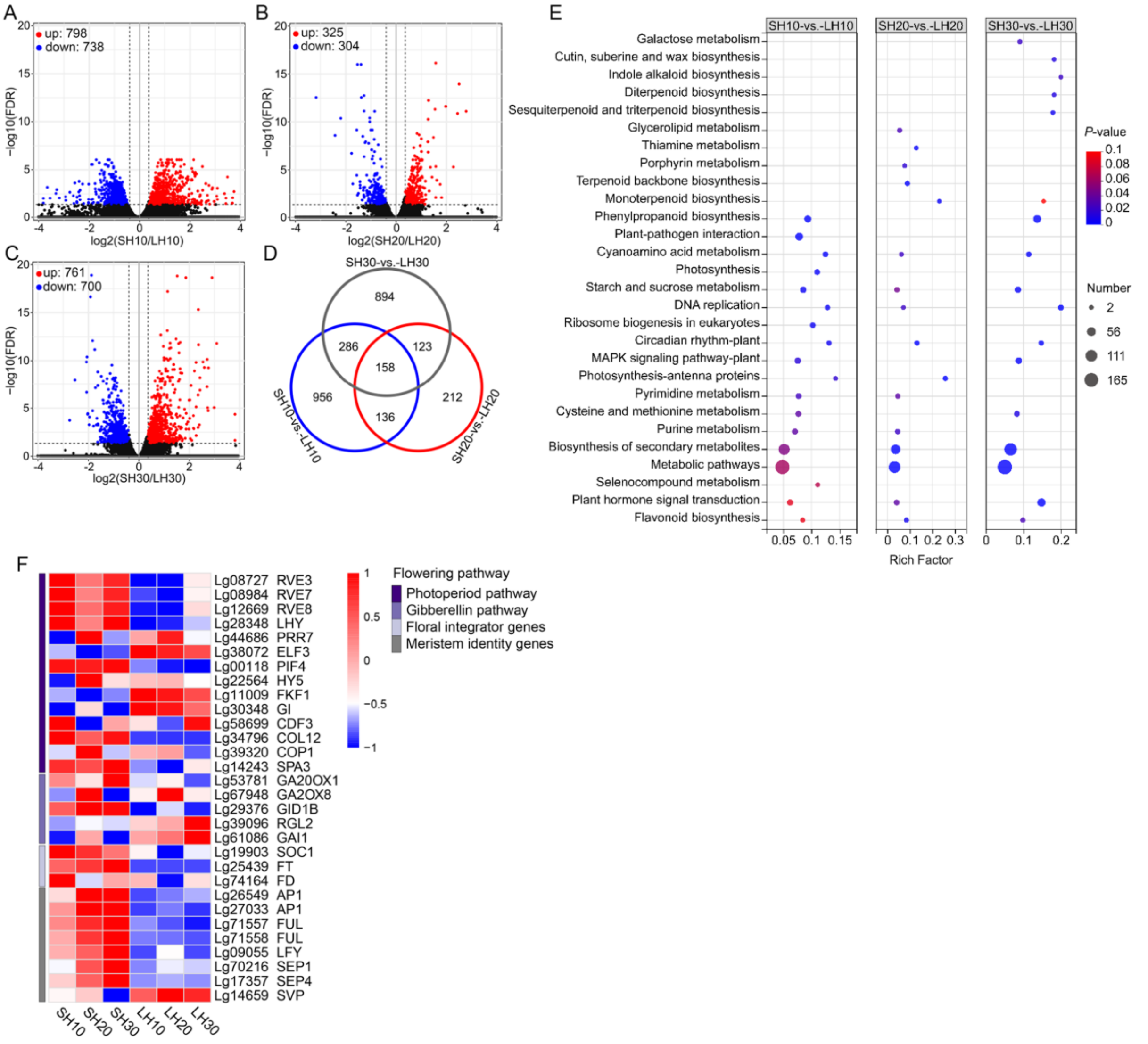

Figure S3. Differentially expressed genes (DEGs) and enrichment analysis. **(A–C)** Number of DEGs across various comparisons: **(A)** SH10 vs. LH10, **(B)** SH20 vs. LH20, and **(C)** SH30 vs. LH30. **(D)** Venn diagram showing differential and stage-specific gene profiles per comparison. **(E)** KEGG pathway enrichment of the DEGs in the three comparisons. The rich factor denotes the ratio of the number of DEGs in a KEGG pathway to the number of all genes annotated to that pathway. **(F)** Heatmap showing expression profiles of the DEGs involved in key flowering pathways in *Luculia gratissima* at 10, 20, and 30 days after the onset of photoperiod treatment. The Z-score normalized RPKM value for an individual gene at a given developmental stage is represented in a blue (low expression) to red (high expression) scale.

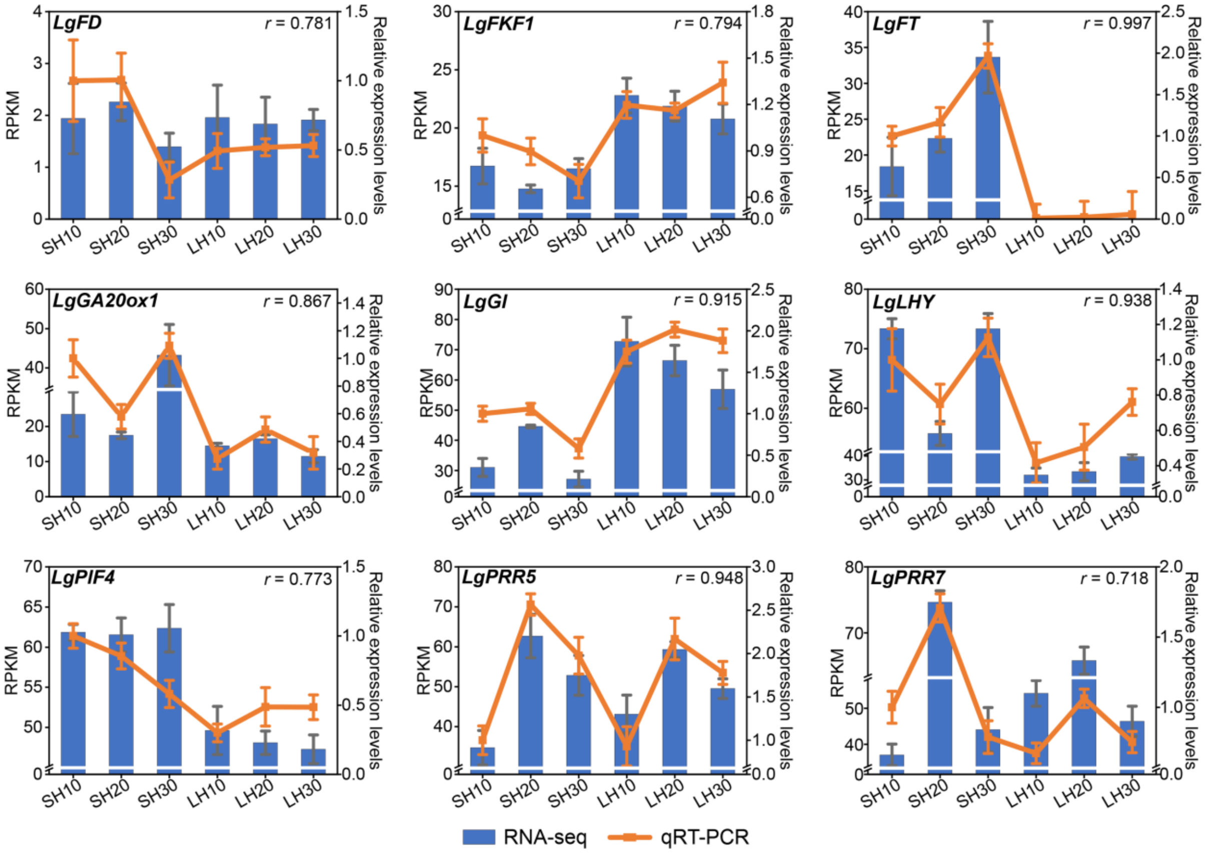

Figure S4. Expression of flowering-related genes at three stages after the onset of photoperiod treatment. Expression levels were analyzed using RNA-seq and confirmed via RT-qPCR. Samples were collected between 10:00 and 10:30 a.m. at 10, 20, and 30 days after initiation of the photoperiod treatment. Data are the mean ± standard error (n = 3). r: Pearson correlation coefficient.

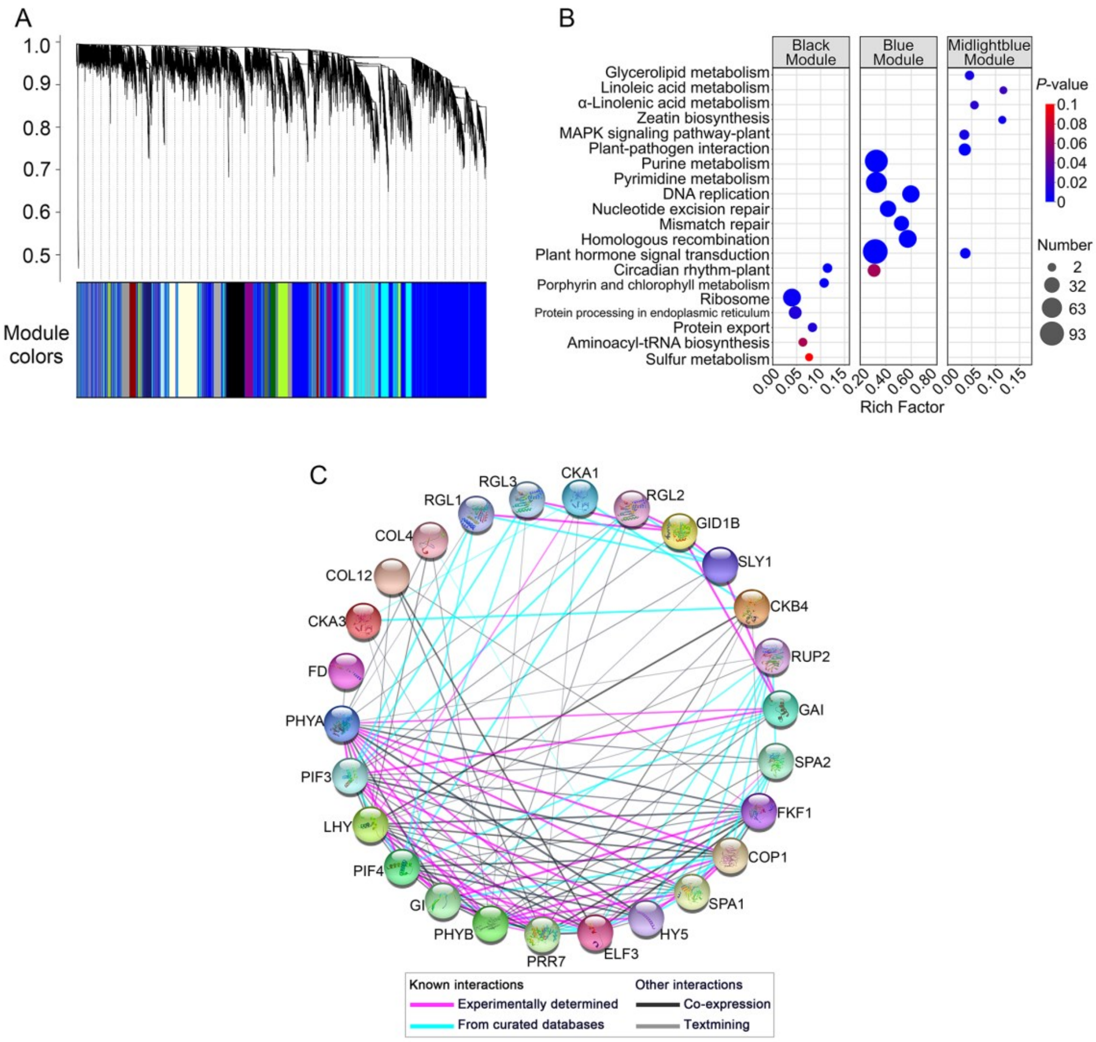

Figure S5. Co-expression regulatory networks based on the weighted gene co-expression network analysis (WGCNA) modules. **(A)** Hierarchical cluster tree showing the co-expression modules. **(B)** KEGG pathway enrichment of the modules black, blue, and midnightblue gene sets. **(C)** STRING-based protein–protein interaction (PPI) network of flowering-related proteins. The different nodes represent different proteins, and the edges indicate potential interaction relationships between the proteins.

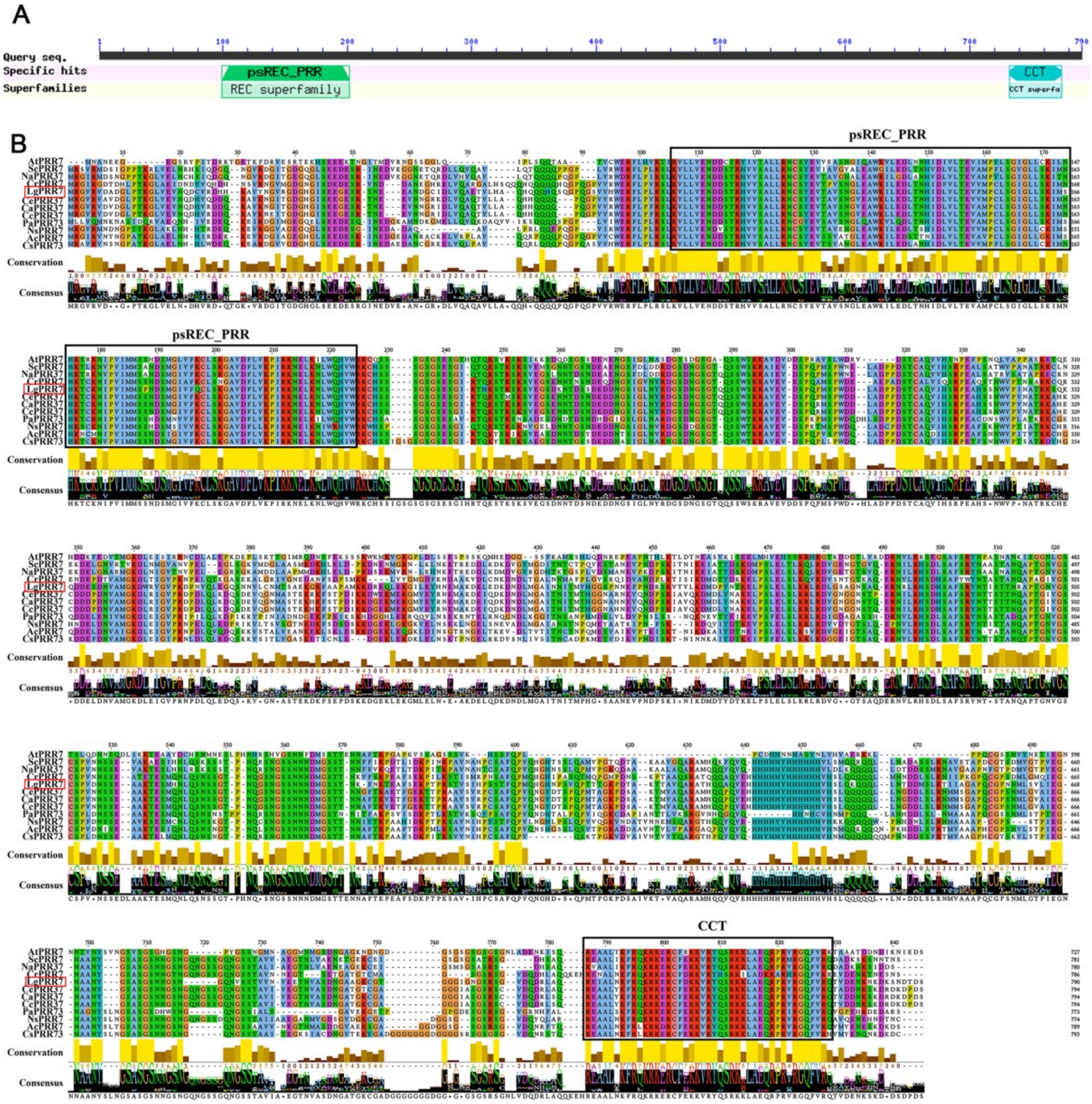

Figure S6. Conserved domains and alignment of LgPRR7 ortholog proteins in different species. **(A)** Prediction of the conserved domain of the LgPRR7 protein in *Luculia gratissima*. **(B)** Alignment of LgPRR7 ortholog proteins in different species. Accession numbers: AtPRR7: NP_568107.1 (At: *Arabidopsis thaliana*); ScPRR7: TMW92515.1 (Sc: *Solanum chilense*); NaPRR37: XP_019246223.1 (Na: *Nicotiana attenuata*); CrPRR7: KAI5648744.1 (Cr: *Catharanthus roseus*); CePRR37: XP_027184967.1 (Ce: *Coffea eugenioides*); CaPRR37: XP_027086994.1 (Ca: *Coffea arabica*); CcPRR37: CDP04335.1 (Cc: *Coffea canephora*); PaPRR73: XP_034919020.1 (Pa: *Populus alba*); NsPRR7: KAA8534314.1 (Ns: *Nyssa sinensis*); AcPRR7: PSS34571.1 (Ac: *Actinidia chinensis*); CsPRR73: XP_028092291.1 (Cs: *Camellia sinensis*).

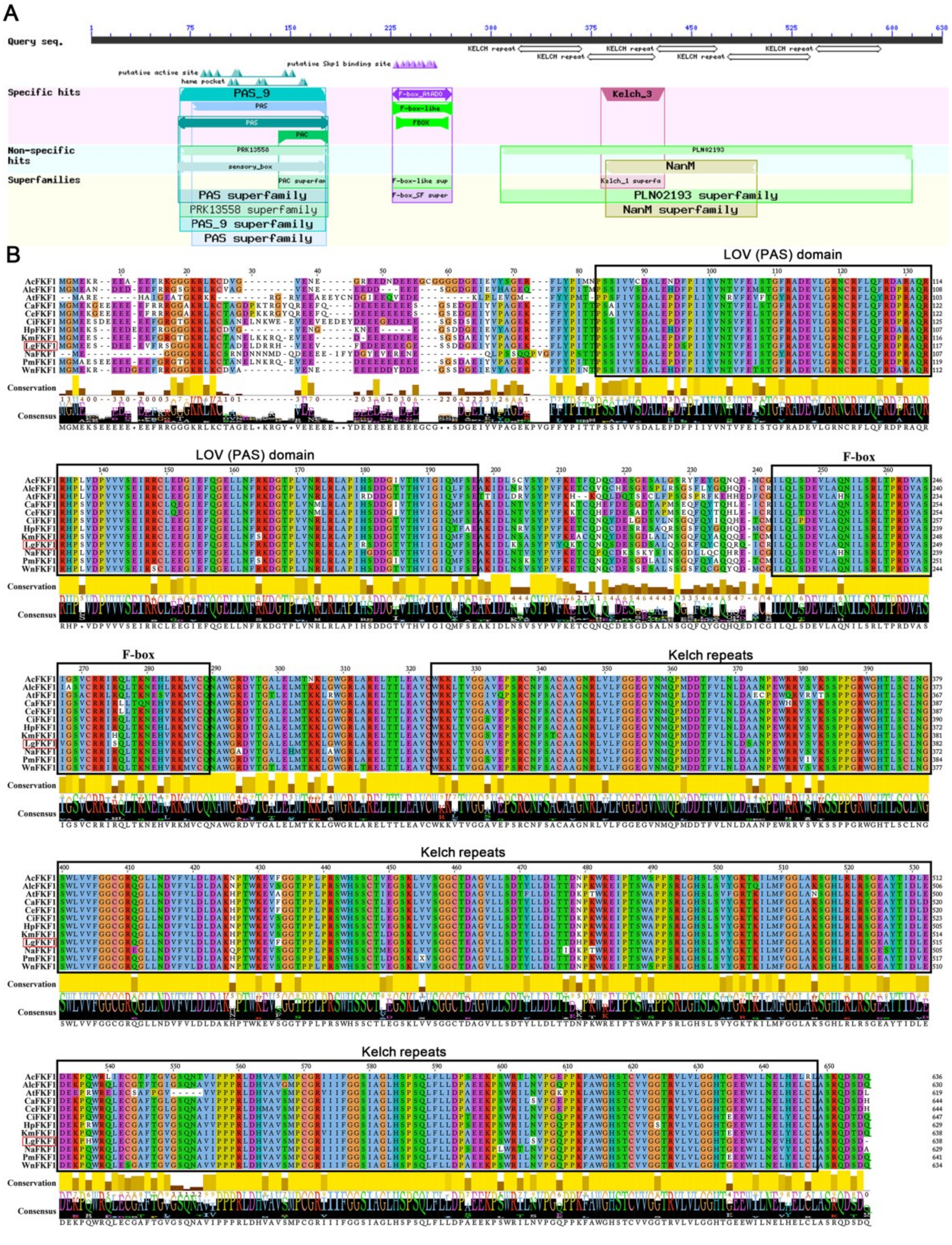

Figure S7. Conserved domains and alignment of LgFKF1 ortholog proteins in different species. **(A)** Prediction of the conserved domain of LgFKF1 protein in *Luculia gratissima.* **(B)** Alignment of LgFKF1 ortholog proteins in different species. Accession numbers: AcFKF1: AML76817.1 (Ac: *Asclepias curassavica*); AlcFKF1: AML77851.1 (Alc: *Allamanda cathartica*); AtFKF1: NP_564919.1 (*Arabidopsis thaliana*); CaFKF1: XP_027098116.1 (Ca: *Coffea arabica*); CeFKF1: XP_027154529.1 (Ce: *Coffea eugenioides*); CiFKF1: AML76527.1 (Ci: *Carapichea ipecacuanha*); HpFKF1: AML77517.1 (Hp: *Holarrhena pubescens*); KmFKF1: AML77708.1 (Km: *Kaliphora madagascariensis*); NaFKF1: AFA35966.1 (Na: *Nicotiana attenuata*); PmFKF1: AML78204.1 (Pm: *Psychotria marginata*); WnFKF1: AML76886.1 (*Wrightia natalensis*).

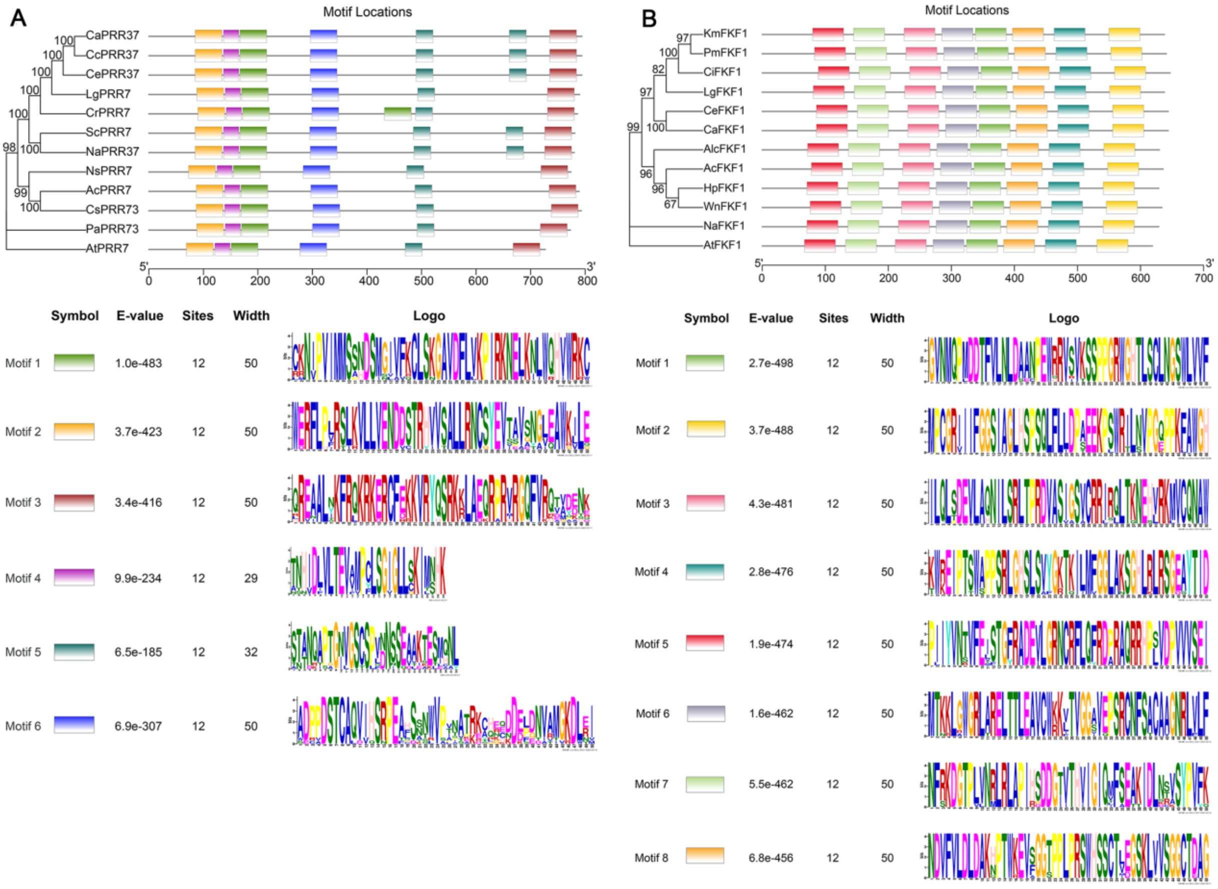

Figure S8. Phylogenetic relationships and conserved motif distribution of LgPRR7 and LgFKF1 ortholog proteins in different species. **(A)** Phylogenetic relationships and conserved motif distribution of LgPRR7 ortholog proteins in different species. **(B)** Phylogenetic relationships and conserved motif distribution of LgFKF1 ortholog proteins in different species. The numbers in the phylogenetic tree (e.g. 97, 99, or 100) represent the bootstrap values.

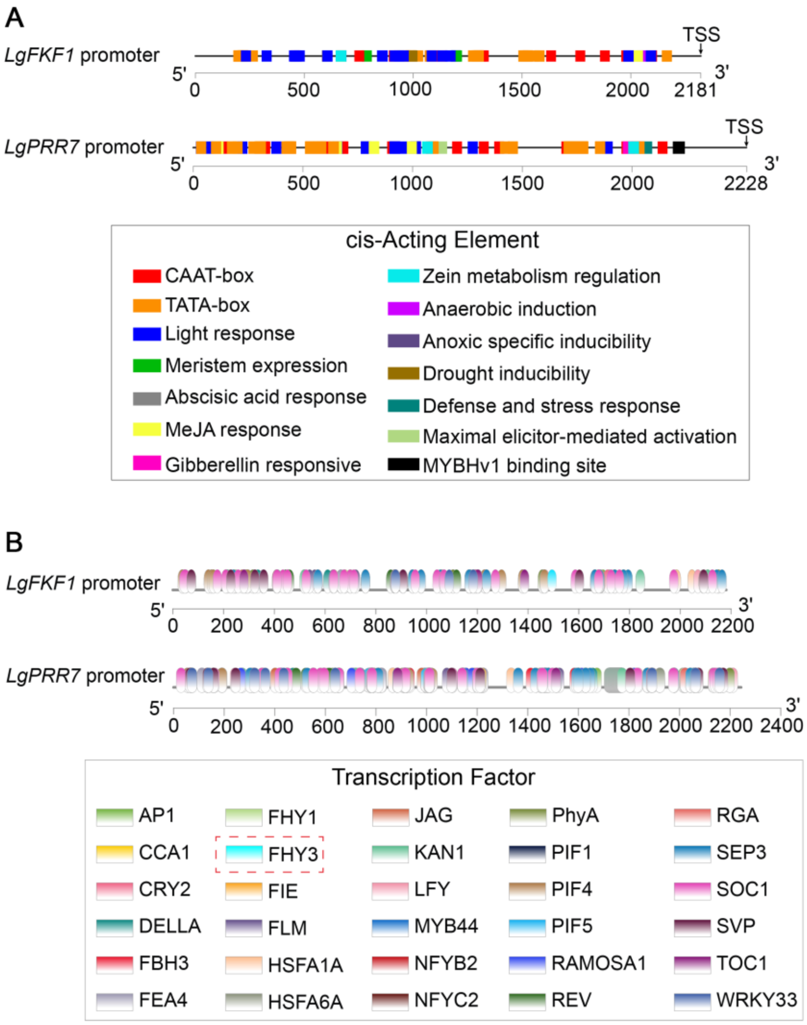

Figure S9. Prediction of *cis*-acting elements and transcription binding sites of *LgPRR7* and *LgFKF1* promoters in *Luculia gratissima.* **(A)** Prediction of *cis*-acting elements of promoter sequences of *LgPRR7* and *LgFKF1* in *L. gratissima.* TSS: Transcription start site. **(B)** Prediction of transcription binding sites of promoter sequences of *LgPRR7* and *LgFKF1* in *L. gratissima.*

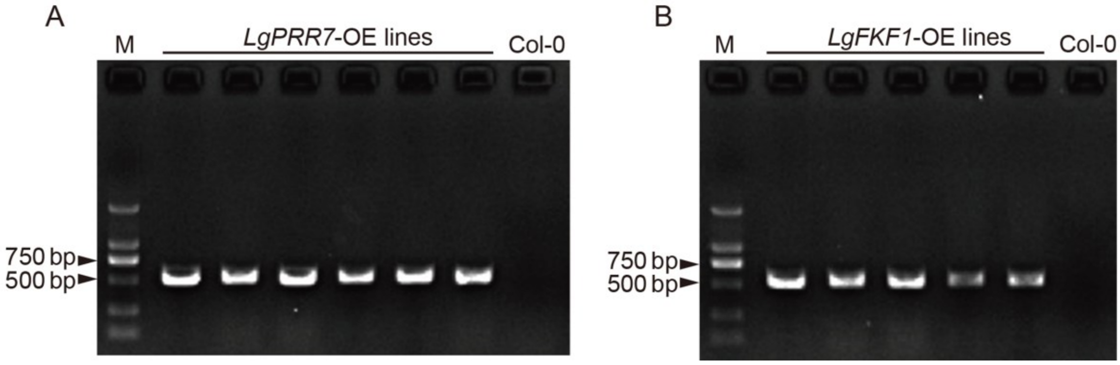

Figure S10. PCR identification of *LgPRR7*- and *LgFKF1*-overexpressing transgenic *Arabidopsis thaliana* lines. **(A)** PCR identification for six independent positive *LgPRR7*-overexpressing transgenic lines. *LgPRR7*-OE lines represent *LgPRR7*-overexpressing transgenic *A. thaliana* lines. **(B)** PCR identification for five independent positive *LgFKF1*-overexpressing transgenic lines. *LgFKF1*-OE lines represent *LgFKF1*-overexpressing transgenic *A. thaliana* lines. The primer pair HPTII-F/R (Table S6) was used to detect positive lines. M: DNA marker.

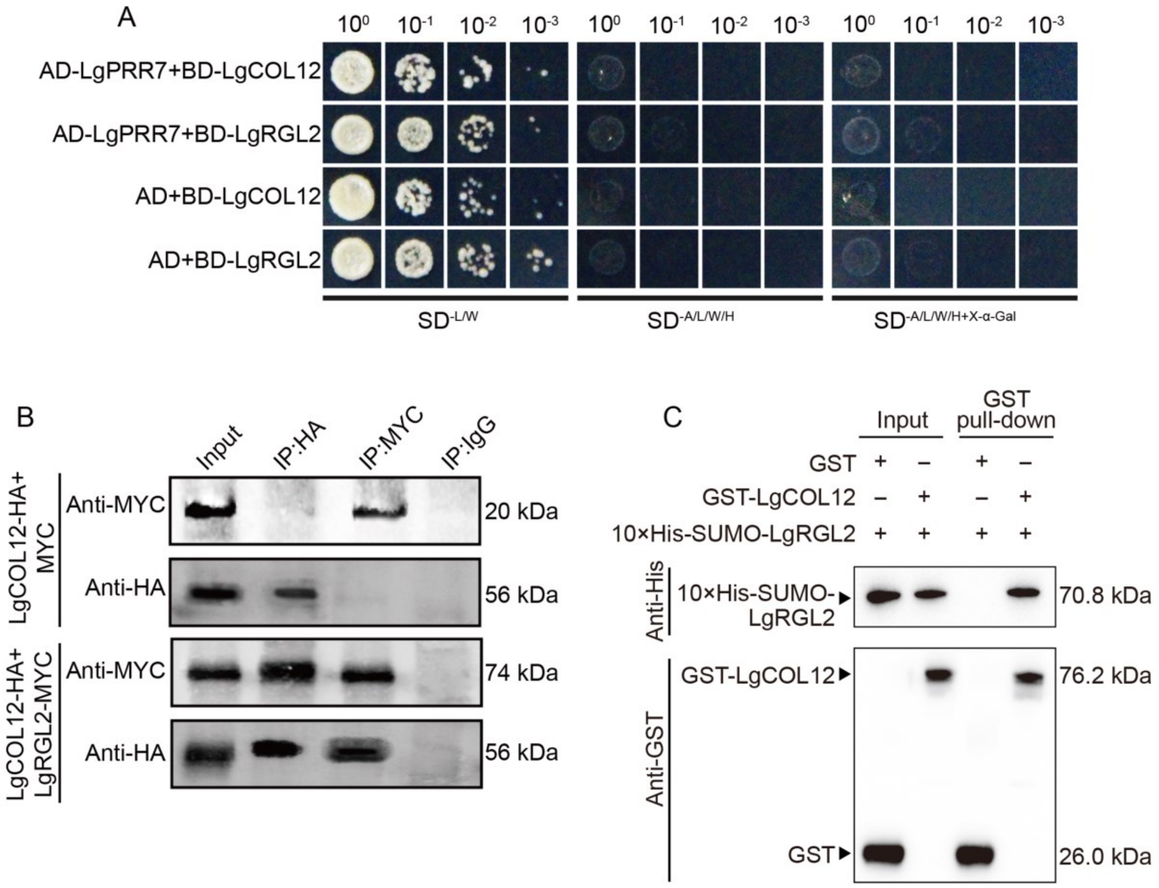

Figure S11. Assessment of the interactions between LgPRR7 and LgRGL2 or LgCOL12, as well as between LgCOL12 and LgRGL2. **(A)** LgPRR7 does not interact with LgCOL12 nor LgRGL2 in yeast, as confirmed using yeast two-hybrid assays. Yeast was spotted on synthetic double dropout (-L/W), quadruple dropout (-A/L/W/H), or quadruple dropout supplemented with X-α-Gal (-A/L/W/H+X-α-Gal) medium. A (Adenine), L (Leucine), W (Tryptophan), H (Histidine). BD, bait protein fused to GAL4 DNA-binding domain; AD, prey protein fused to GAL4 activation domain. The combinations pGADT7-T+pGBKT7-53 and pGADT7-T+ pGBKT7-lam were used as the positive and negative controls, respectively. **(B)** Co-immunoprecipitation (Co-IP) assay showing that LgCOL12 interacts with LgRGL2 in tobacco leaves. Protein samples were extracted from tobacco leaves transiently expressing LgRGL2-MYC and LgCOL12-HA. Immunoprecipitates obtained with anti-MYC or anti-HA magnetic microbeads were analyzed using immunoblotting with anti-MYC and anti-HA antibodies, respectively. Input (crude extracts) and IgG antibody were used as positive and negative controls, respectively. **(C)** *In vitro* pull-down assay showing the direct interaction of LgCOL12 and LgRGL2. Recombinant 10×His-SUMO-LgRGL2 was incubated with immunoprecipitated GST-LgCOL12. Proteins were detected using immunoblotting with anti-His or anti-GST antibodies. The "+" or "–" represent the presence or absence of proteins, respectively.

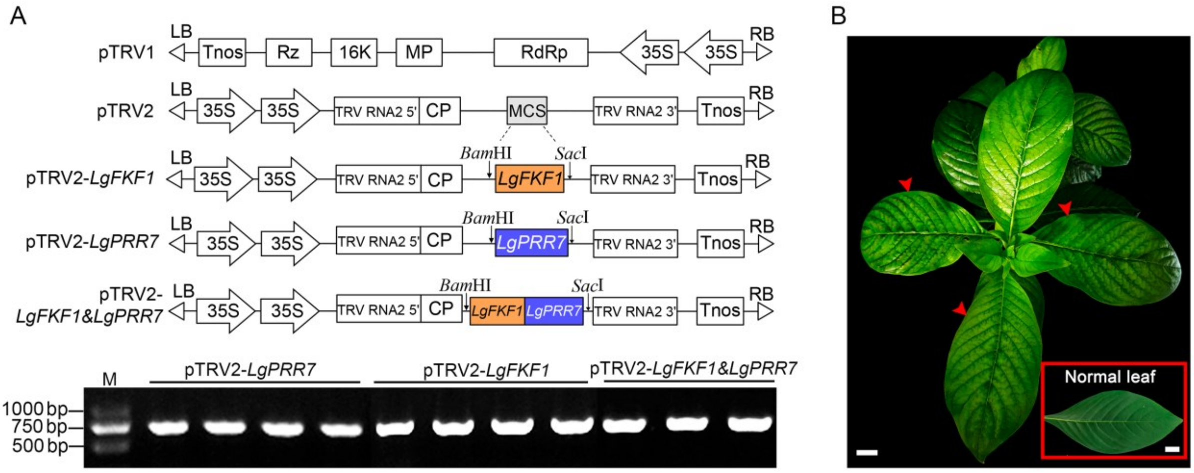

Figure S12. Identification *LgPRR7*- and *LgFKF1*-silenced vectors. **(A)** Structure diagrams of pTRV2 recombinant vectors and PCR identification of colonies containing recombinant pTRV2 vectors. **(B)** Phenotypes of pTRV-*NbPDS* *Luculia gratissima* plants. Scale bar: 1 cm.
